# Rapid ^14^C excursion at 3372-3371 BCE not observed at two different locations

**DOI:** 10.1038/s41467-020-20695-y

**Published:** 2021-01-29

**Authors:** A. J. Timothy Jull, Irina P. Panyushkina, Mihály Molnár, Tamás Varga, Lukas Wacker, Nicolas Brehm, Elemér Laszló, Chris Baisan, Matthew W. Salzer, Willy Tegel

**Affiliations:** 1 Department of Geosciences, University of Arizona, Tucson, AZ USA; 2 Isotope Climatology and Environmental Research Centre, Institute for Nuclear Research, Debrecen, Hungary; 3 Laboratory for Tree-Ring Research, University of Arizona, Tucson, AZ USA; 4 Laboratory of Ion Beam Physics, ETH-Zürich, Zürich, Switzerland; 5 Department of Forest Growth and Dendroecology, University of Freiburg, Freiburg, Germany

**Keywords:** Solar physics, High-energy astrophysics

**Arising from** Wang et al. *Nature Communications* 10.1038/s41467-017-01698-8 (2017)

Excursions in the carbon-14 (^14^C) record measured in tree rings are attributed to various high energy but short-lived cosmic effects^1–7^. Wang et al.^8^ proposed a new event at 3372–3371 BCE based on a single set of annual ^14^C data measured on a floating tree rings from a buried specimen of Chinese wingnut (*Pterocarya stenoptera*). Here we attempt to reproduce this event in tree rings of an absolutely dated bristlecone pine specimen (*Pinus longaeva*) from the White Mountains, USA and a subfossil oak (*Quercus* sp.) from the Moselle River Valley, France. We cannot confirm the presence of a cosmic-ray event as suggested at 3372–3371 BCE and we discuss potential implications to earlier results.

Carbon-14 (half-life 5730 years) is produced by the reaction of secondary thermal neutrons derived from the primary cosmic-ray flux on nitrogen^9^. This ^14^C is incorporated into the terrestrial carbon cycle within 1–2 years as is most convincingly demonstrated through the^14^C signal of anthropogenic nuclear testing^10^. Miyake et al.^2–4^ were the first to study the annual signal of ^14^C in tree rings to reveal annual excursions, which were much larger than those observable in the decadally resolved international radiocarbon calibration curve (IntCal20)^11^. Specifically, they observed spikes in ^14^C activity at 774–775 CE and 993–994 CE^2,4^. The 774 CE event was first confirmed in a bristlecone pine record^4^, and indeed, this work encouraged many subsequent studies looking for both these events and searches for other events. Büntgen et al.^5^ summarizes ^14^C series of trees for 774–775 CE from 34 locations and another set of trees for 993–994 CE from 10 locations around the globe. Another rapid event at 660 BCE has been reproduced in different records and is therefore widely accepted^7,12,13^. Separately, other rapid changes that may show more complex solar dynamo phenomena or combinations of solar and galactic events at 5480 BCE and 813 BCE have been observed^14,15^.

An important task in demonstrating a convincing global signature of a ^14^C excursion is to reproduce the event with tree rings from different geographic locations. We endeavored to reproduce the ^14^C sequence of Wang et al.^8^ using two independent tree-ring records. First, we measured ^14^C on tree rings of bristlecone pine from the White Mountains in California. This tree species is well known for a remarkably long lifespan often exceeding several thousand years. The SH146-2003 remnant specimen was collected at the Sheep Mountain high-elevation site and rigorously cross-dated with the site master chronology spanning 4408 BCE–2014 CE^16^. Second, we developed ^14^C tree-ring series from subfossil oak collected from a gravel pit near Champey-sur-Moselle. The CHEY1-17 oak specimen is absolutely cross-dated with the South German oak chronology covering 8240 BCE–2017 CE^17^.

We obtained ^14^C measurements on tree rings from a bristlecone pine for the interval 3351–3392 BCE (42 years) and from an oak for 3350–3390 BCE (41 years). These ^14^C series are developed from a 328-year specimen of bristlecone pine (3598–3271 BCE) and a 93-year oak tree (3402–3310 BCE), where the rings are absolutely dated via cross-dating with original site (master) chronologies. The Pearson^18^ correlation (*R*) between the tree-ring width series of samples SH146-2003 and CHEY1-17 and their master chronologies is 0.57 (probability, *p* < 0.01) and 0.52 (*p* < 0.01), respectively. This experiment allows us to observe if the Wang et al.^8^ event occurred during the same sampling period of 3358–3388 BCE.

Figure 1 and Supplementary Table 1 present our results in a Δ^14^C plot^19^ against known age of the tree rings. A 11-year periodicity with an amplitude of ~5 per mil on a declining trend of Δ^14^C^18^ has clearly appeared. We note that our new two ^14^C series (pine and oak) are well correlated (*R* = 0.89). Student’s *t* test^18^ of these two data sets shows good agreement with *t* = -0.69 (*p* = 0.49, critical value Tc = 1.99). We compared our series to those of Wang et al.^8^. Intriguingly, the Chinese wingnut ^14^C measurements agree with the general trend of our results, except for the 2 years of 3370–3371 BCE, where Wang et al.^8^ reported the new ^14^C excursion, and the year 3388 BCE. The excursion proposed by Wang et al.^8^ cannot be confirmed in the other two ^14^C sets. To confirm that the records are statistically different, we performed *t* test on each set of data. Convincingly, the hypothesis fails *t* test with *t* = −2.15 (*p* = 0.038, Tc = 2.02). Because the Chinese wingnut specimen was dated with ^14^C wiggle-matching, we developed longer ^14^C series covering two decades prior to 3371 BCE where the calendar dates might shift due to wiggle-matching. Nevertheless, we do not observe any excursion consistent with Wang et al.^8^.

**Fig. 1:**
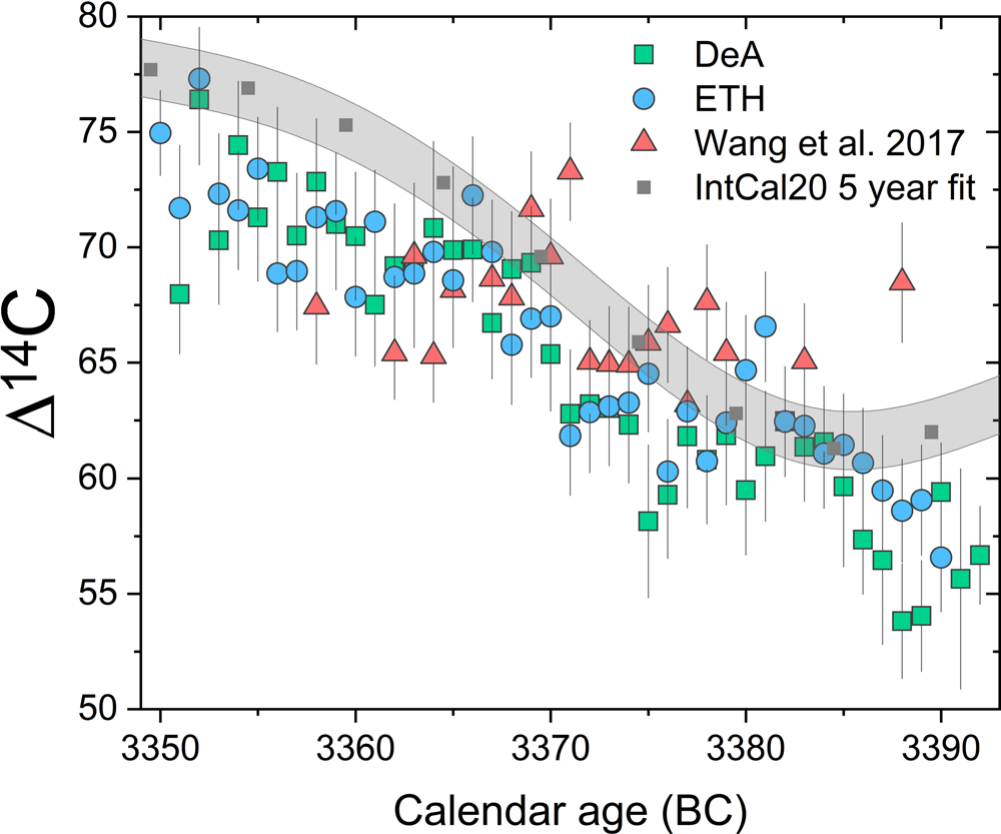
Measurements of Δ^14^C (in ‰) vs. dendrochronologically derived calendar age for samples of bristlecone pine (measured in Debrecen, DeA) and European Oak (measured in Zürich, ETH), compared to the reported Δ^14^C results for Chinese wingnut of Wang et al.^8^. In the case of the wingnut samples, the age reported by ref. ^8^ is used as the age on the ordinate axis. The green squares show data from Debrecen, the blue circles data from ETH, and the red triangles show the results of Wang et al.^8^. The gray area shows the international calibration curve (IntCal20)^11^ with 1*σ* error, which is based on a spline fit to the IntCal20 at 5-year resolution.

Our results raise some important points regarding radiocarbon and dendrochronological dating. Wang et al.^8^ state that they used traditional cross-dating and correlated the wingnut rings with a master chronology of tree-ring widths from California downloaded from the International Tree-Ring Data Bank. We assume that it was a 7091-year record (5142 BCE–1962 CE) of bristlecone pine. However, the dating approach applied by Wang et al.^8^ is far from conventional dendrochronology. The annual ring variations of bristlecone pine from the alpine environments are limited by the cold and extremely dry climate^20^. The pine growth is not comparable to Chinese wingnut, which has a completely different ecological amplitude, different climatic controls, and grows ~10,000 km away. Application of cross-dating to wingnut species is very limited due to interannual density fluctuations and/or missing rings. Besides, the wingnut specimen contained only ~60 rings. Further, Wang et al.^8^ used Dendrochronology Program Library, a software package not used to measure rings. We believe that it is important for cross-dating to be carried out according to well-established methods and practices^21^.

The wingnut specimen was ^14^C dated prior to the spike study of high-resolution ^14^C measurements. As described^8^, the wingnut rings were counted, and four 5-year groups sampled for a wiggle-matching test. Wiggle-matching technique ties knowingly spaced-age differences (e.g., ring groups) via a Monte Carlo simulation of a chi-squared fit between ^14^C data and the IntCal curve^13,22^. The 4-point sequence of 5-year groups fitted to IntCal mean^8^ is not reliable. The ^14^C variation observed by^8^ could deviate from their assigned age by several decades or more. We re-simulated the wingnut fit^8^ using the D-Sequence function of OxCal^22^ that placed the older end of the wingnut sequence to 3496-3458 BCE rather than the 3388 BCE. We observe an apparent 11-year solar cycle in our data as shown in a wavelet analysis of Fig. 2 and Supplementary Fig. 1. This cycle shows a ^14^C variation up to ~5 per mil over one solar cycle. This is somewhat larger than that observed in recent trees^9^, although similar to the solar-cycle effects observed by Jull et al.^15^.

**Fig. 2:**
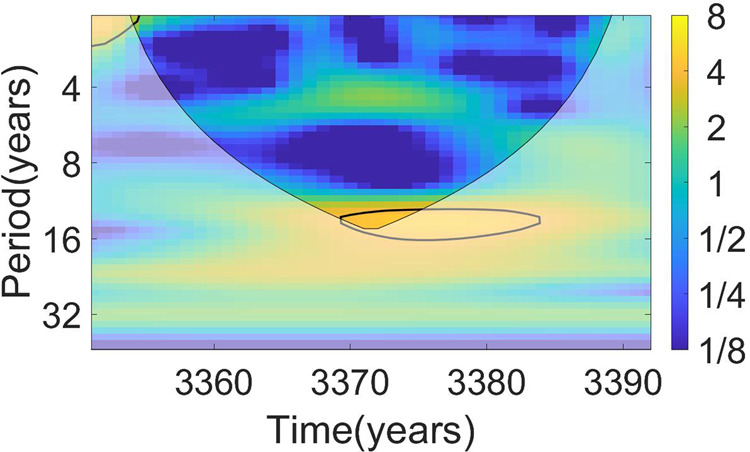
Wavelet analysis of the bristlecone pine ^14^C series. Shades denote the dimensionless continuous wavelet transform power-spectrum density. The color bar placed on the right represents the squared power of the wavelet transform and the numbers indicate the spectrum power level (dimensionless). High values in a few time intervals (*x*-axis) with some periodicity (*y*-axis) show that the amplitude of a signal with that periodicity emerges from noise in those years. The black contour denotes the 5% significance level against red noise. The area where edge effect influence distorts the picture is shown in lighter shades. The signal attributable to solar the solar cycle is marked with yellow fields. The black line outlines the variance significant relative to red noise. A similar result can be obtained for the European Oak series (shown in Supplementary Fig. 1).

Our two independently derived records do not confirm the ^14^C event at 3372–3371 BCE described by Wang et al.^8^. Therefore, unless this can be independently confirmed in other trees or other proxy records for cosmogenic isotopes^12,13^, we recommend excluding this result from any list of Solar Proton Events. We also conclude that the wingnut rings are not from the interval 3350–3390 BCE. The result of Wang et al.^8^. may either be at a different time period or may be due to unaccounted effects that have not been fully evaluated. Since our results are generally consistent with the trend of Wang et al.’s^8^ data with the exception of the 2 years (3370 and 3371 BCE) and 1 other year (3388 BCE), we may also conclude that the result is actually in general agreement, but that errors in Wang et al.^8^, who used two different laboratories, have been underestimated.

## Methods

The bristlecone wood specimen used in this study (Supplementary Fig. 2) was collected by M. Salzer in July 2003 at the elevation of 3575 m asl on the Sheep Mountain ridge (37.53475N and 118.20045W). The ring widths of this specimen were measured on a Lintab system. The 328-year ring series is correlated with the 6422-year master chronology from Sheep Mountain spanning from 4408 BCE to 2014 CE, which is composed of tree rings from remnant wood and overlaps with rings from living trees^16^. The Sheep Mountain chronology also correlates well with two other long bristlecone tree-ring chronologies from the same mountain range confirming the dating accuracy of the chronology. Dating SH146-2003 was assigned using correlation of ring widths between 50-year segments and the master SHP mountain chronology lagged successively by 25 years with statistical verification using COFECHA12K version 6.06P, which uses multiple parametric statistics to examine the quality of various fits^20^. Second checks on dating were confirmed through the presence/absence of sub-annual ring features, such as frost rings. Further details of master chronology construction are published in refs. ^16,20^. The cross-dating and the master chronology development were performed at the Laboratory of Tree-Ring Research, University of Arizona. The SH146-2003 remnant specimen has no locally absent rings and includes a very prominent frost ring formed in 3458 BCE that is also present in the same year in one other sample of the master chronology. The annual growth layers were separated through cutting with a razor blade and grounded to 20-μm mesh. Each powdered sample was converted to α-cellulose using standard procedures^23,24^.

The oak sample (CHEY1-17) originated from an accumulation of 17 subfossil oak trunks found during gravel extraction in the Moselle River valley (48.9405N, 6.0586E) in 2011 (Supplementary Fig. 3). Tree-ring widths were measured to an accuracy of 1/100 mm using a stereo microscope and a measuring system. The annually resolved tree-ring width series of 14 tree trunks are synchronized into a 247-year mean chronology. This site chronology is overlapped with the master oak chronology for South Germany^17^. After 10-year smoothing spline transformation, these two chronologies correlate at *R* = 0.53 for the common period from 3405 BCE to 3159 BCE. The cross-dating was performed at University of Freiburg.

Bristlecone cellulose samples were combusted to CO_2_ and converted to graphite, and ^14^C dating was performed using the 200 kV MICADAS (Mini Carbon Dating System, Ion Plus AG, Dietikon, Switzerland) at the Institute for Nuclear Research in Debrecen, Hungary^23^. Sample calculation and data reduction were done using the standard BATS software^25^. Oak samples were converted to α-cellulose using the procedures described in ref. ^24^, and the accelerator mass spectrometer (AMS) samples were converted to graphite using the automated graphitization equipment (AGE-3) system at the Swiss Federal Institute of Technology, Zürich. Samples were measured on a MICADAS 200 kV AMS of the same design and software^25^ as the Debrecen machine.

## Supplementary information

Supplementary Information

## Data Availability

Tree-ring sample information can be found in the International Tree-Ring Databank https://www.ncdc.noaa.gov/data-access/paleoclimatology-data/datasets/tree-ring. The bristlecone pine information is given in https://www.ncdc.noaa.gov/paleo-search/study/3254. All data are public information accessible from the authors on request.

## Code availability

Wiggle-matching can be undertaken using OxCal (https://c14.arch.ox.ac.uk/oxcal.html) using the international radiocarbon calibration curves. Dendrochronology Program Library is available at https://www.ltrr.arizona.edu/software.html. The COFECHA code can be downloaded from https://www.ldeo.columbia.edu/tree-ring-laboratory/resources/software.
