# Publisher Correction: Rapid ^14^C excursion at 3372-3371 BCE not observed at two different locations

**DOI:** 10.1038/s41467-021-21647-w

**Published:** 2021-02-23

**Authors:** A. J. Timothy Jull, Irina P. Panyushkina, Mihály Molnár, Tamás Varga, Lukas Wacker, Nicolas Brehm, Elemér Laszló, Chris Baisan, Matthew W. Salzer, Willy Tegel

**Affiliations:** 1 Department of Geosciences, University of Arizona, Tucson, AZ USA; 2 Isotope Climatology and Environmental Research Centre, Institute for Nuclear Research, Debrecen, Hungary; 3 Laboratory for Tree-Ring Research, University of Arizona, Tucson, AZ USA; 4 Laboratory of Ion Beam Physics, ETH-Zürich, Zürich, Switzerland; 5 Department of Forest Growth and Dendroecology, University of Freiburg, Freiburg, Germany

**Keywords:** Solar physics, High-energy astrophysics

Correction to: *Nature Communications* 10.1038/s41467-020-20695-y, published online 29 January 2021.

The original HTML version of this Article was updated shortly after publication because the previous HTML version linked to an incorrect Supplementary information file.

## Supplementary information

Supplementary information

