# Supplementary material for: Rapid 14C excursion at 3372-3371 BCE not observed at two different locations
Source: Nat Commun. 2021 Jan 29;12:712. doi: 10.1038/s41467-020-20695-y (PMC7846558; doi:10.1038/s41467-020-20695-y)
Supplement: Supplementary file 1 — Supplementary Information [file 41467_2020_20695_MOESM1_ESM.pdf]

Supplementary Information

Surface-bound reactive oxygen species generating nanozymes for selective antibacterial action

Feng Gao, Tianyi Shao, Yunpeng Yu, Yujie Xiong*, and Lihua Yang*

ADDITIONAL RESULTS AND DISCUSSION

1. Environmental pH imposes negligible effects on $AgPd_{0.38}$'s ROS Production.

To examine whether environmental pH affects $AgPd_{0.38}$'s ROS production, we firstly need to select an ROS probe that functions at different pH. Ascorbic acid (AA) is an antioxidant[1] whose strong absorption at 266 nm disappears upon oxidation[2]. 9,10-anthracenediyl-bis(methylene) dimalonic acid (ABDA) is a fluorescent molecule but upon capturing $^1O_2$ becomes non-fluorescent[3]. Singlet oxygen sensor green (SOSG) is weakly blue fluorescent but upon capturing $^1O_2$ becomes brightly green fluorescent[4]. When environmental solution pH was varied from 1 to 12, AA's absorbance (at 266 nm) and SOSG's fluorescence emission spectrum both changed significantly (Supplementary Fig. 8a-c). Indeed, AA's absorbance (at 266 nm) and SOSG's fluorescence (520-600 nm) disappeared when pH $\geq$ 11 and $\leq$ 3, respectively. In similar assays, ABDA's fluorescence was affected by change in environmental pH but remained brightly fluorescent across the whole examined pH range (Supplementary Figure 8b). Therefore, we used ABDA as the ROS probe for evaluating the effect of pH on $AgPd_{0.38}$'s ROS production.

Briefly, we incubated $AgPd_{0.38}$ in ABDA-containing PBS buffer at 37 ºC for 3 h and then recorded ABDA's fluorescence spectrum ($\lambda_{ex}/\lambda_{em}$ = 380 nm / 400-600 nm) with a fluorimeter (Supplementary Fig. 8d-o). For $AgPd_{0.38}$-treated ABDA at each pH, we included ABDA treated similarly but with PBS at a same pH as a reference, in efforts to exclude the intrinsic influence of environmental pH on ABDA's fluorescence. We found that the relative fluorescence intensity of $AgPd_{0.38}$-treated ABDA to its

corresponding PBS-treated counterpart was barely affected by change in environmental pH (Supplementary Fig. 8p), suggesting negligible effects of pH on $AgPd_{0.38}$'s ROS production.

2. Environmental temperature imposes negligible effects on $AgPd_{0.38}$'s ROS Production.

To examine whether environmental temperature affects $AgPd_{0.38}$'s ROS production, we firstly need to select an ROS probe that functions at different temperature. As environmental temperature was changed (4-55 ºC), AA's absorbance was influenced significantly (Supplementary Fig. 9a) while the fluorescence emission spectra of ABDA and SOSG were almost unaffected (Supplementary Fig. 9b-c). Therefore, we selected ABDA and SOSG as the ROS probes for evaluating the effects of environmental temperature on $AgPd_{0.38}$'s ROS production.

Briefly, we incubated $AgPd_{0.38}$ in ABDA-containing PBS buffer for 3 h at a specified temperature (4, 15, 25, 37, 45, or 55 ºC) and then recorded the fluorescence emission spectrum of ABDA ($\lambda_{ex}/\lambda_{em}$ = 380 nm / 400-600 nm) with a fluorimeter (Supplementary Fig. 9d-i). For $AgPd_{0.38}$-treated ABDA at each temperature, ABDA treated similarly but with PBS at a same temperature was included as a reference, to exclude the intrinsic (though slight) influrence of temperature on ABDA's fluorescence. We found that the relative fluorescence intensity (at 433 nm) of $AgPd_{0.38}$-treated ABDA to its corresponding PBS-treated counterpart was barely affected by change in environmental temperature (Supplementary Fig. 9j). Similar results were observed when replacing ABDA with SOSG (Supplementary Fig.10). Collectively, these results suggests

negligible effects of environmental temperature on $AgPd_{0.38}$'s ROS production.

3. Buffer agent imposes negligible effects on $AgPd_{0.38}$'s ROS Production.

To examine whether buffer agent affects $AgPd_{0.38}$'s ROS production, we used AA as an ROS probe and incubated $AgPd_{0.38}$ with AA (at 37 ºC, for 3 h) in different buffers (at pH = 7.4), followed by recording AA's absorption spectrum with an UV-vis spectrometer (Supplementary Fig. 11a-d). For $AgPd_{0.38}$-treated AA in each buffer, AA treated similarly but with the same buffer (*i.e.*, without $AgPd_{0.38}$) was included as a reference, to exclude potential influence of buffer agent on AA's absorbance. We found that the relative absorbance of $AgPd_{0.38}$-treated AA to its corresponding buffer-treated counterpart was negligibly affected by the change in buffer agent (Supplementary Fig. 11a-d), suggesting negligible effects of buffer agent on $AgPd_{0.38}$'s ROS production. Consistently, assays performed similarly but with ABDA ($\lambda_{ex}/\lambda_{em}$ = 380 nm/400-600 nm) as the ROS probe (Supplementary Fig. 11e-h) showed that change in buffer agent barely affected the relative fluorescence intensity (at 433 nm) of $AgPd_{0.38}$-treated ABDA to its corresponding buffer-treated counterpart (Supplementary Fig. 11i). Clearly, change in buffer agent imposes negligible effects on $AgPd_{0.38}$'s ROS production.

4. A lipid bilayer is permeable to free $^1O_2$.

Chlorin e6 (Ce6) is an organic photosensitizer that generates free $^1O_2$ upon light irradiation ($\lambda \sim$ 660 nm)[5, 6]. To examine whether a lipid bilayer is permeable to free $^1O_2$, we coated Ce6-preloaded PLGA (poly(lactic-co-glycolic acid)) nanoparticle (Ce6/PLGA) with a lipid bilayer (DOPC:DSPE-PEG = 0.90:0.10) (Fig. 1f) and used the resulting Ce6/PLGA@lipid particle (Fig. 1g and Supplementary Fig. 17) as a model

for nanoparticles that produce free $^1O_2$. Specifically, dispersion of Ce6/PLGA@lipid in SOSG-containing PBS was irradiated with a solar simulator (at 0.1 W/m^2, 5-min) and then submitted to recording on the fluorescence emission spectrum of SOSG ($\lambda_{ex}/\lambda_{em}$ = 504 nm/510-700 nm), with that of Ce6/PLGA in SOSG-containing PBS included for comparison. Control is nanoparticle-absent PBS that contains same dose of SOSG. Our results (Fig. 1h) show that the fluorescence intensity of SOSG in light-irradiated Ce6/PLGA@lipid dispersion was significantly higher than that of SOSG in light-irradiated PBS, indicative of SOSG oxidation to appreciable extent in response to $^1O_2$ generated by Ce6 upon light irradiation. Of note, the fluorescence emission spectrum of SOSG in Ce6/PLGA@lipid dispersion was almost identical to that of SOSG in Ce6/PLGA dispersion (Fig.1h), indicative of comparable extent of SOSG oxidation by $^1O_2$, suggesting negligible retardance on the efflux of free $^1O_2$ and/or consumption of free $^1O_2$ by the lipid bilayer coating in Ce6/PLGA@lipid. In short, a lipid bilayer is permeable to free $^1O_2$.

5. PMDS wafer was successfully coated with $AgPd_{0.38}$.

We used a PDMS wafer as the model for biomedical device surfaces and coated it successively with dopamine[7, 8] and $AgPd_{0.38}$ (Figure 5a and Supplementary Figure 52-53), by taking advantage of the presence of poly(vinylpyrrolidone) (PVP) over $AgPd_{0.38}$ and PVP's ability to form hydrogen bonds[7, 8] and electrostatic interaction[7, 8] with polydopamine (PDA). The dopamine-modified intermediate wafer and the final $AgPd_{0.38}$-modified wafer were named PDMS/PDA and PDMS/PDA/AgPd, respectively.

Unlike the pristine PDMS wafer, PDMS/PDA exhibited three peaks at 3400, 1613 and 1510 cm^{-1} (Supplementary Fig. 53b), which are attributable to PDA, indicative of successful polymerization of dopamine on the PDMS wafer and attachment of the resulting PDA thereon. Moreover, under SEM, both PDMS/PDA and PDMS/PDA/AgPd exhibited a rather rough surface with frequent appearance of wrinkles whereas the pristine PDMS surface appeared rather smooth (Fig. 5e, 1st row), indicative of successful surface modification. In addition, the water contact angles of the pristine PDMS, the intermediate PDMS/PDA, and the final PDMS/PDA/AgPd were found to be 104.8°, 87.9° and 56.3°, respectively (Supplementary Fig. 53c). Collectively, these results suggest that the pristine PDMS wafer was modifed successively and successfully with dopamine and $AgPd_{0.38}$. The average $AgPd_{0.38}$ concentration on the final PDMS/PDA/AgPd was further quantified to be 11.23 ± 1.40 $\mu g/cm^2$ with inductively-coupled plasma mass spectrometry (ICP-MS).

ADDITIONAL TABLES

Supplementary Table 1. Nanozymes previously reported in the literature[9-40].

Nanomaterial	Enzyme-mimetic activity	Cytotoxicity (IC_{50}[a])	Antibacterial potency (MBC_{99}[b])	References
Ag	Peroxidase	—[c]	>7 ppm	9
GQD/AgNP	Oxidase	—	>2 μg/mL	10
GQDs	Peroxidase	—	>100 μg/mL	11
MoS_2	Peroxidase	—	150 μg/mL	12
MOF/GO_x	Peroxidase	—	>100 μg/mL	13
Cu_2WS_4	Oxidase/peroxidase	—	1.7 μg/mL	14
Pd@Pt-T790	Peroxidase	—	—	15
CeO_{2-x}	Haloperoxidase	—	—	16
MSN-AuNPs	Oxidase/peroxidase	—	>800 μg/mL	17
PMCS	Peroxidase	—	—	18
MOF-Au-Ce	Peroxidase	—	—	19
Au/C_3N_4	Peroxidase	100 μg/mL	>20 μg/mL	20
CuO-HCSs	Peroxidase	—	250 μg/mL	21
CNTs	Peroxidase	—	>100 μg/mL	22
Pt/Ag	Oxidase/peroxidase	—	>10 μg/mL	23
V_2O_5	Haloperoxidases	—	—	24
Pd	Oxidase/peroxidase	—	>14.7 μg/mL	25
SAF NCs	Peroxidase	—	62.5 μg/mL	26
UsAuNPs	Peroxidase	—	>1 mg/mL	27
PCN-224-Pt	catalase	12.5 μg/mL	—	28
PMR	catalase	25 μg/mL	—	29
AMP	peroxidase	50 μg/mL	—	30
IMSN-PEG-TI	peroxidase/catalase	100 μg/mL	—	31
DMSN-Au-Fe_3O_4	Oxidase/peroxidase/catalase	100 μg/mL	—	32
PtFe@Fe_3O_4	Oxidase/peroxidase	50 μg/mL	—	33
CoO@AuPt	Oxidase/peroxidase/catalase	200 μg/mL	—	34
ZIF@GO_x/GQDs	peroxidase	12.5 μg/mL	—	35
Fe/Al-GNEs	peroxidase	20 μg/mL	—	36
FA-PNCNzymes@IAA	peroxidase	10 μg/mL	—	37
Pt-carbon	catalase	100 μg/mL	—	38
OxgeMCC-rSAE	catalase	4 ppm	—	39
MnO_2@PtCo	oxidase/catalase	50 μg/mL	—	40

[a] IC_{50}, the minimum concentration to kill 50% of inoculated mammalian cells. [b] MBC_{99}, the minimum concentration to kill 99% of inoculated bacterial cells. [c] Not reported.

ADDITIONAL FIGURES

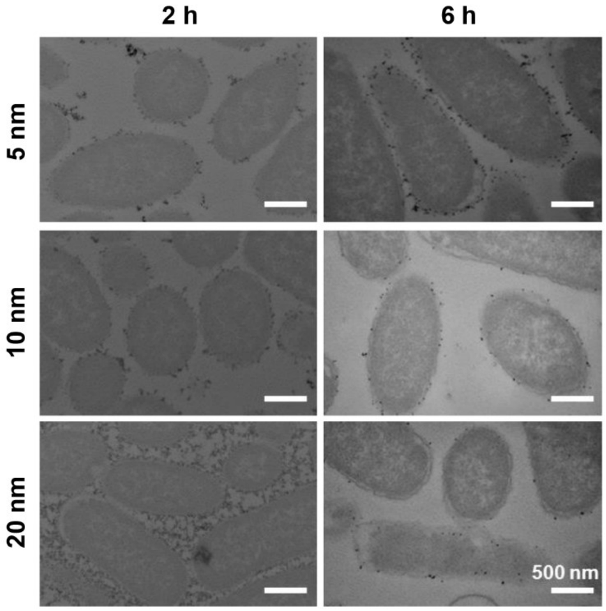

Supplementary Figure 1. TEM images of *E. coli* cells after 2- or 6-h co-incubation with gold nanoparticles (50 μg/mL) of different sizes (5 nm, 10 nm, or 20 nm) in PBS.

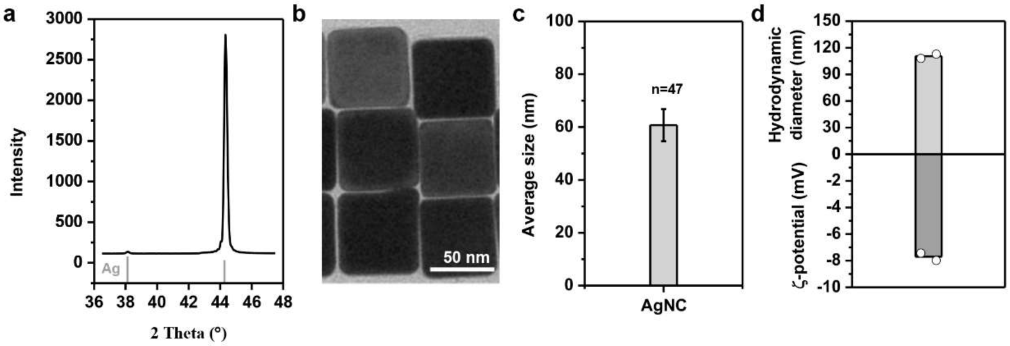

Supplementary Figure 2. Characterizations on the precursor Ag nanocube (AgNC). (a) XRD spectrum and (b) TEM image of AgNC. (c) Average size of AgNC. Data points are reported as mean ± standard deviation (n = 47 individual nanocubes in TEM

images). (d) Hydrodynamic diameter and surface zeta-potential (ζ-potential) of AgNC (10 μg/mL) in Millipore water.

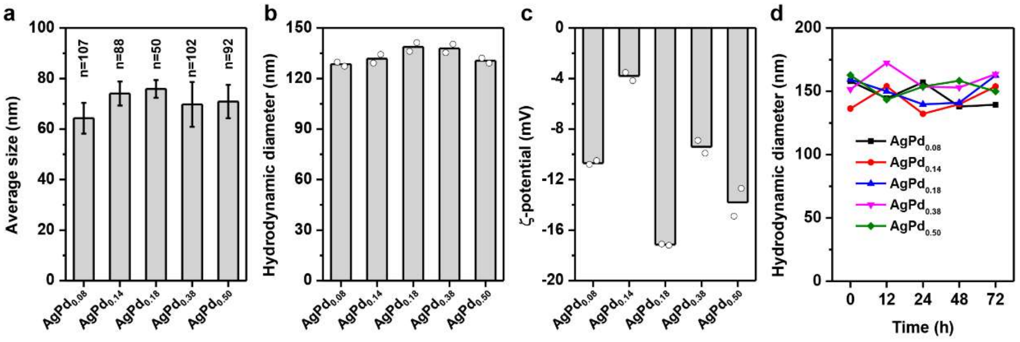

Supplementary Figure 3. Characterizations on the AgPd nanocages. (a) Average sizes of AgPd nanocages, averaged over "n" individual nanoparticles in their respective TEM images. (b) Hydrodynamic diameters and (c) ζ-potentials of AgPd nanocages (10 μg/mL) in Millipore water. (d) Hydrodynamic diameters of AgPd nanocages in PBS over a span of 72 hours.

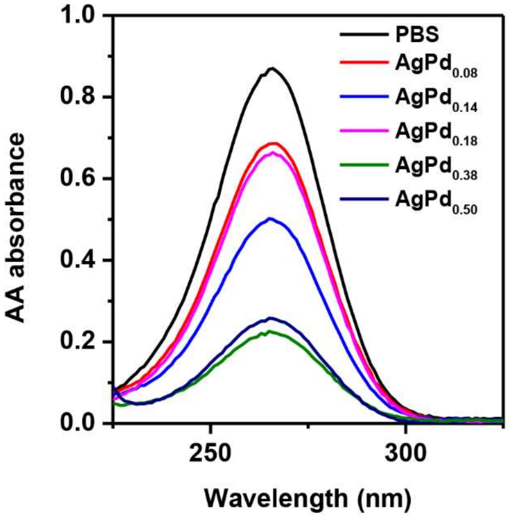

Supplementary Figure 4. ROS generation by the AgPd nanocages. The absorption

spectra of ascorbic acid (AA) after 3-h treatment with an AgPd nanocage (8 μg/mL) in PBS, with that of AA treated similarly but with PBS included as a control.

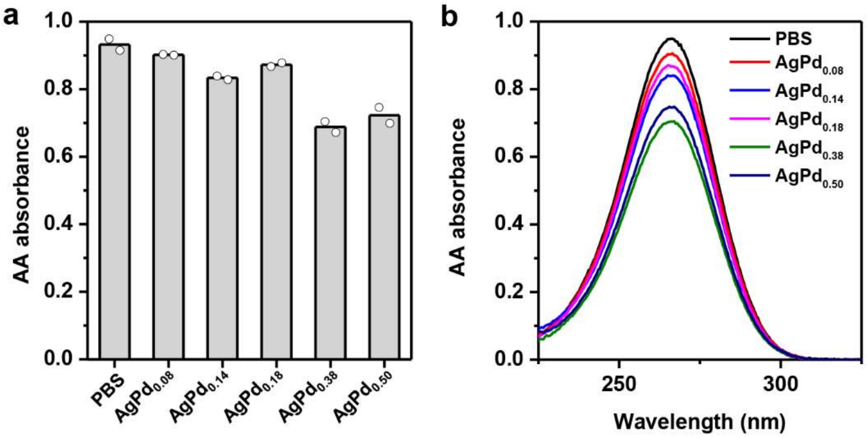

Supplementary Figure 5. ROS generation by the AgPd nanocages. (a) The absorbance (λ = 266 nm) and (b) absorption spectra of AA after 1-h treatment with AgPd nanocages (8 μg/mL). AA treated similarly but with PBS was included as a control.

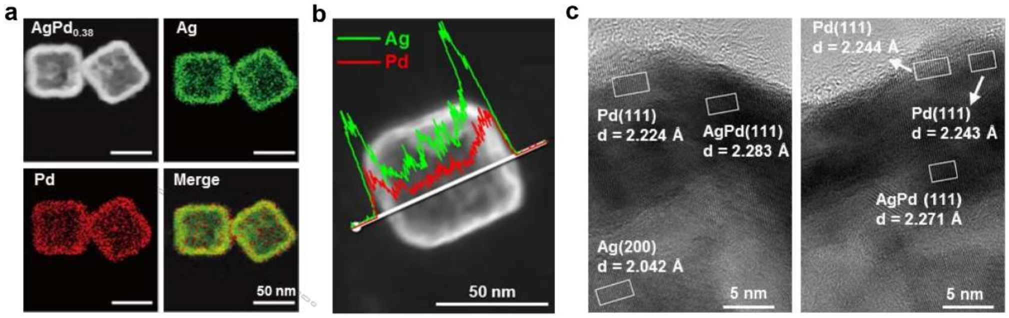

Supplementary Figure 6. Characterizations on $AgPd_{0.38}$. (a) Elemental mapping profiles of $AgPd_{0.38}$ under energy dispersive spectroscopy (EDS), in which red and green signals indicate Pd and Ag elements, respectively. (b) EDS line scan profiles of an individual $AgPd_{0.38}$ particle, in which red and green lines indicate Pd and Ag signals,

respectively. (c) High resolution TEM images showing two selected areas of $AgPd_{0.38}$.

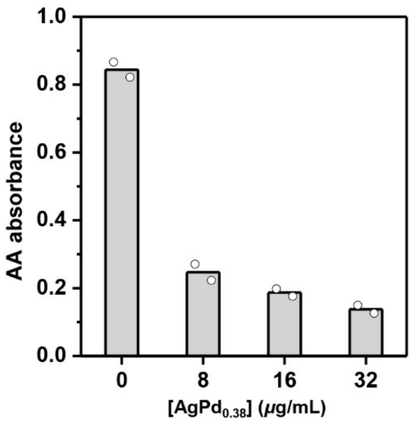

Supplementary Figure 7. Dose-dependence in the ROS generation by $AgPd_{0.38}$. The absorbance (λ = 266 nm) of AA after 3-h treatment with $AgPd_{0.38}$ at differing concentration (0, 8, 16, and 32 μg/mL).

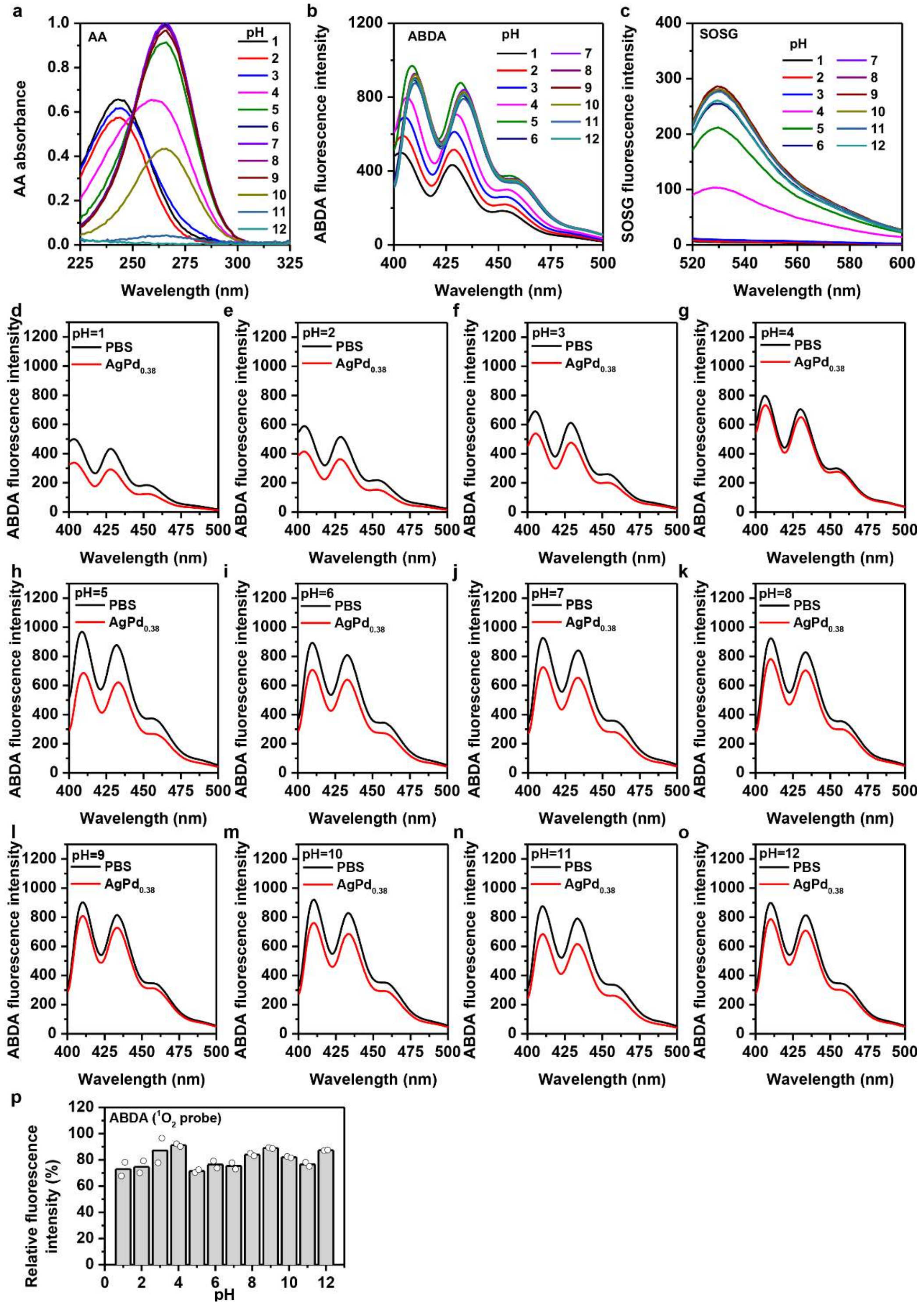

Supplementary Figure 8. Effects of pH on ROS generation by $AgPd_{0.38}$. (a) Absorption spectra of AA and (b-c) fluorescence emission spectra of (b) ABDA and (c) SOSG after 3-h incubation in PBS at different pH. (d-o) Fluorescence emission spectra

of ABDA after 3-h treatment with $AgPd_{0.38}$ (64 μg/mL) in PBS at different pH, with those of ABDA treated similarly but with PBS included as controls. (p) Relative fluorescence intensity of $AgPd_{0.38}$-treated ABDA to its corresponding PBS-treated counterpart.

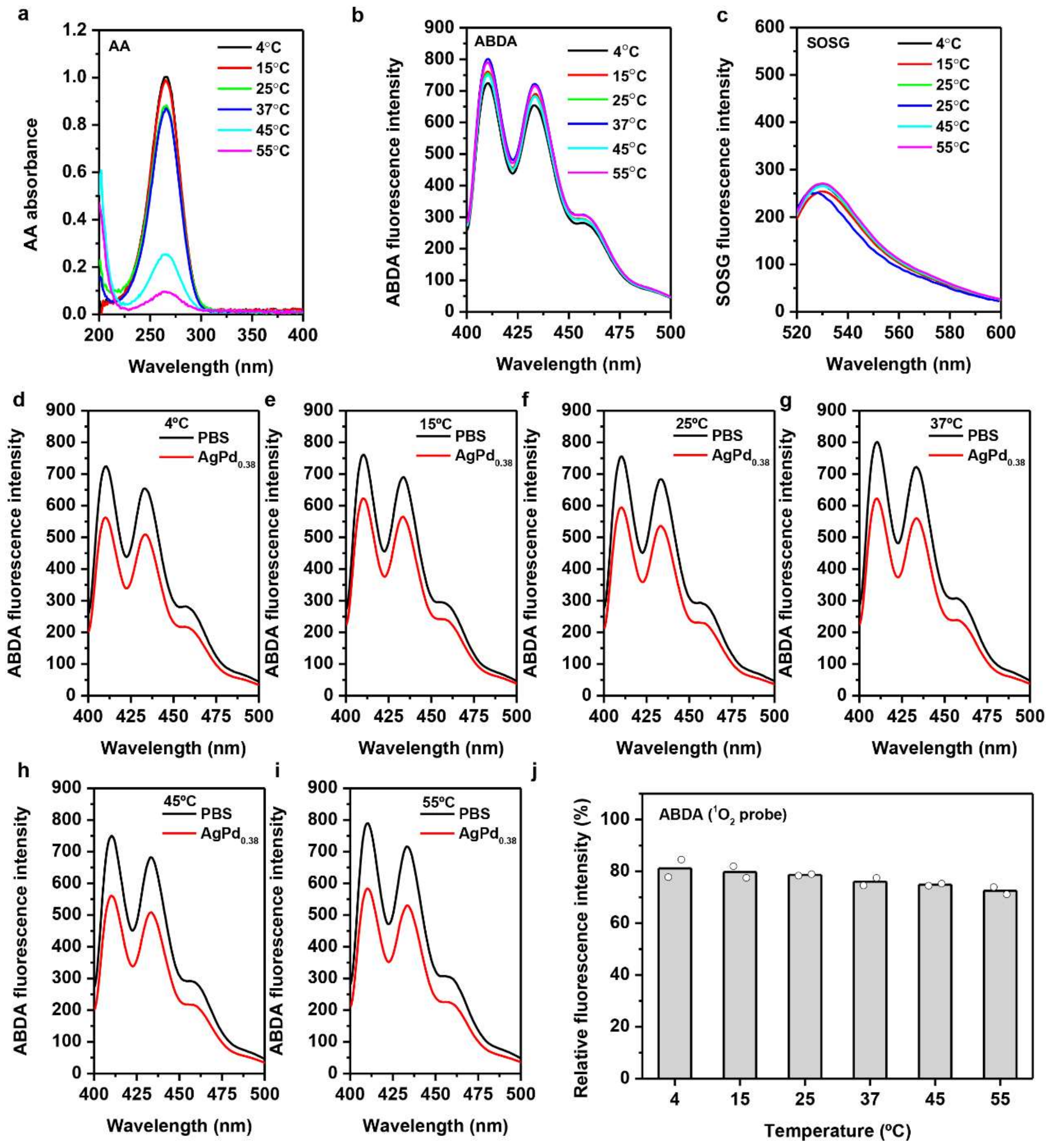

Supplementary Figure 9. Effects of temperature on ROS generateion by $AgPd_{0.38}$ using AA as the ROS probe. (a) Absorption spectra of AA and (b-c) fluorescence

emission spectra of ABDA and (c) SOSG after 3-h incubation in PBS at different temperature. (d-i) Fluorescence emission spectra of ABDA after 3-h treatment with $AgPd_{0.38}$ (64 μg/mL) in PBS at different temperature, with those of ABDA treated similarly but with PBS included as controls. (j) Relative fluorescence intensity of $AgPd_{0.38}$-treated ABDA to its corresponding PBS-treated counterpart.

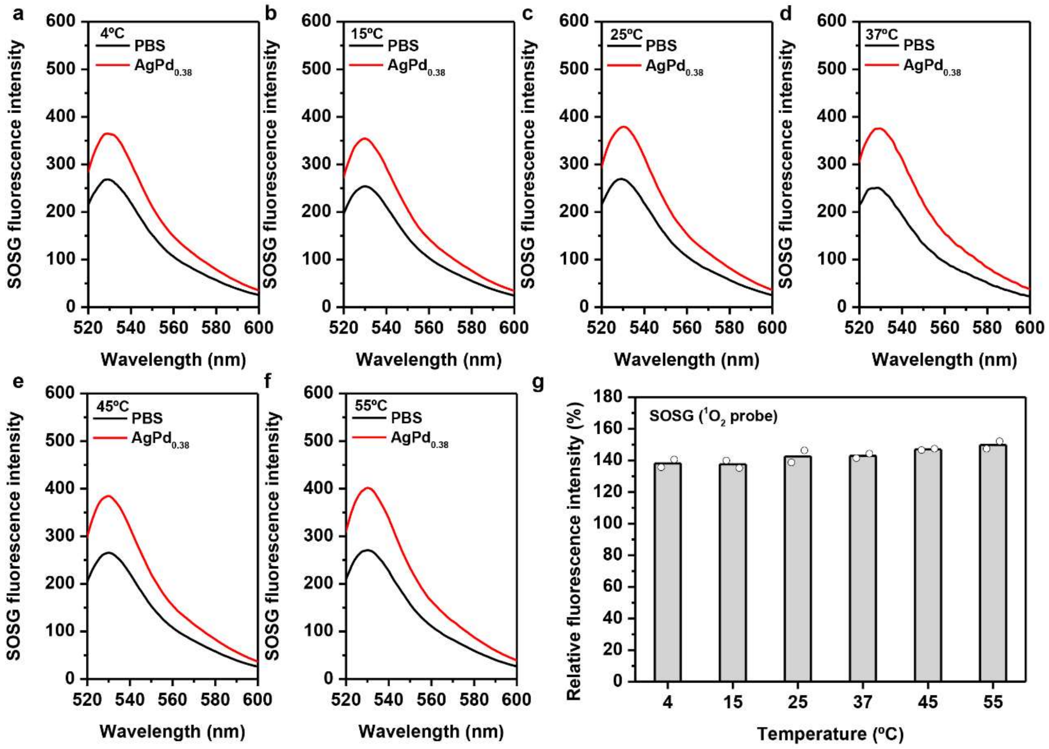

Supplementary Figure 10. Effects of temperature on ROS generation by $AgPd_{0.38}$, using SOSG as the ROS probe. (a-f) Fluorescence emission spectra of SOSG after 3-h treatment with $AgPd_{0.38}$ (64 μg/mL) in PBS at different temperature, with those of SOSG treated similarly but with PBS included as controls. (g) Relative fluorescence intensity of $AgPd_{0.38}$-treated SOSG to its corresponding PBS-treated counterpart.

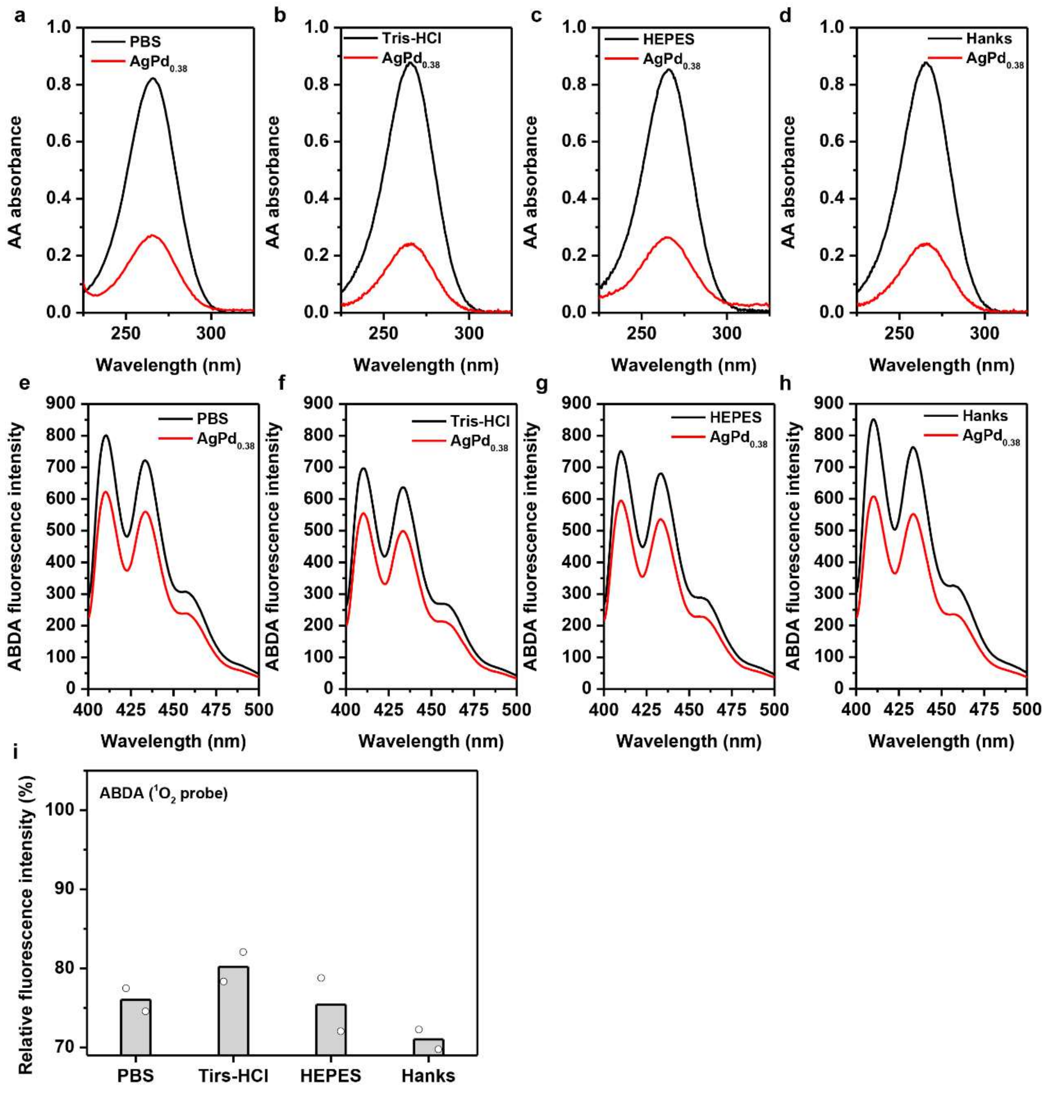

Supplementary Figure 11. Effects of buffer agent on ROS generation by $AgPd_{0.38}$. (a-d) Absorption spectra of AA after 3-h treatment with $AgPd_{0.38}$ (8 μg/mL) in different buffers, with those of AA treated similarly but with the corresponding buffers included as controls. (e-g) Fluorescence emission spectra of ABDA after 3-h treatment with $AgPd_{0.38}$ (64 μg/mL) in different buffers, with those of ABDA treated similarly but with the corresponding buffers included as controls. Buffers used here include PBS, Tris-HCl, HEPES, and Hanks, and the pH of these buffers was set to be constant at 7.4. (i) Relative fluorescence intensity of $AgPd_{0.38}$-treated ABDA to its corresponding buffer-

treated counterpart.

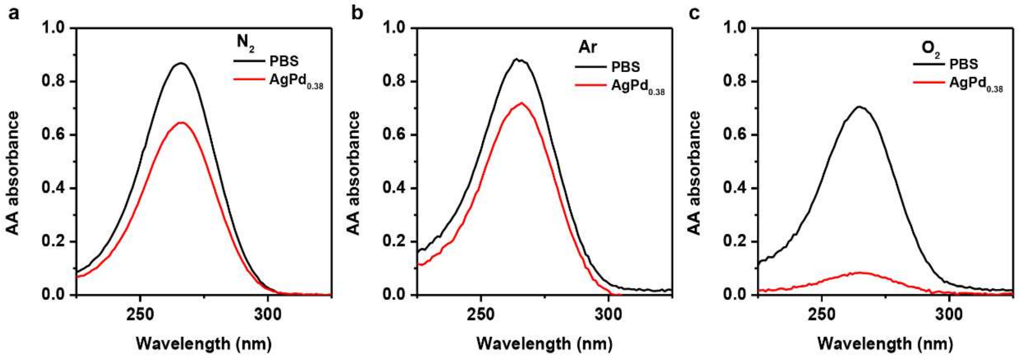

Supplementary Figure 12. ROS generation by $AgPd_{0.38}$ requires O_2, but neither N2 nor Ar. Absorption spectra of AA after 3-h treatment with $AgPd_{0.38}$ (8 μg/mL) in PBS purgered with (a) N_2, (b) Ar and (c) O_2. Those of AA treated similarly but without $AgPd_{0.38}$ (*i.e.*, with PBS) were included as controls.

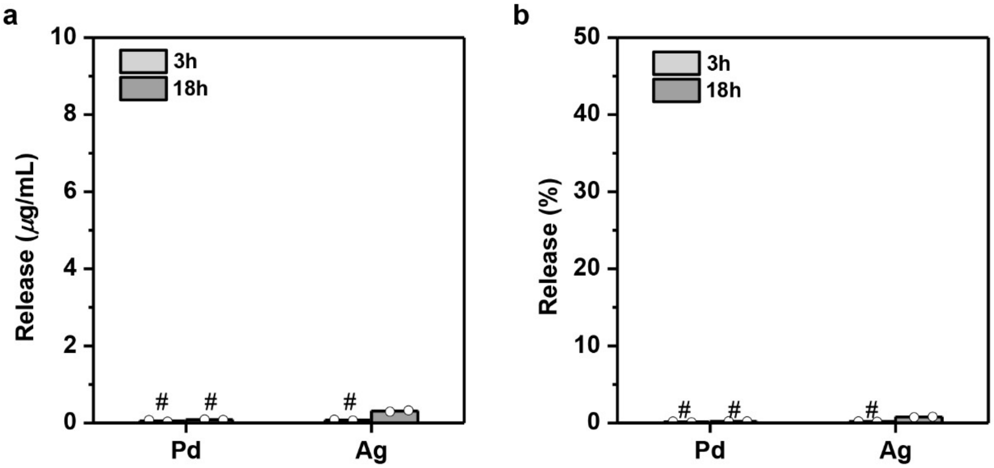

Supplementary Figure 13. Absence of metal ion release from $AgPd_{0.38}$. (a) The concentrations of Pd and Ag elements in the environmental solutions and (b) their relative percentages to the initial amount of $AgPd_{0.38}$ after 3- and 18-h incubation of $AgPd_{0.38}$ (40 μg/mL) in PBS at 37 ºC. # in (a) indicates concentrations ≤ 0.08 μg/mL. #

in (b) indicates percentage of ≤ 0.2%.

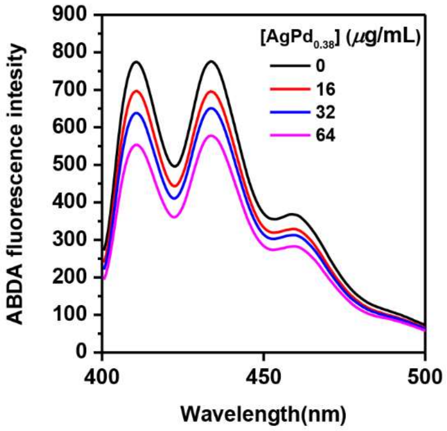

Supplementary Figure 14. Oxidation of ABDA by the ROS generated by $AgPd_{0.38}$. (a) Fluorescence emission spectra of ABDA after 3-h treatment with $AgPd_{0.38}$ at differing concentrations (0, 16, 32, and 64 μg/mL) in PBS.

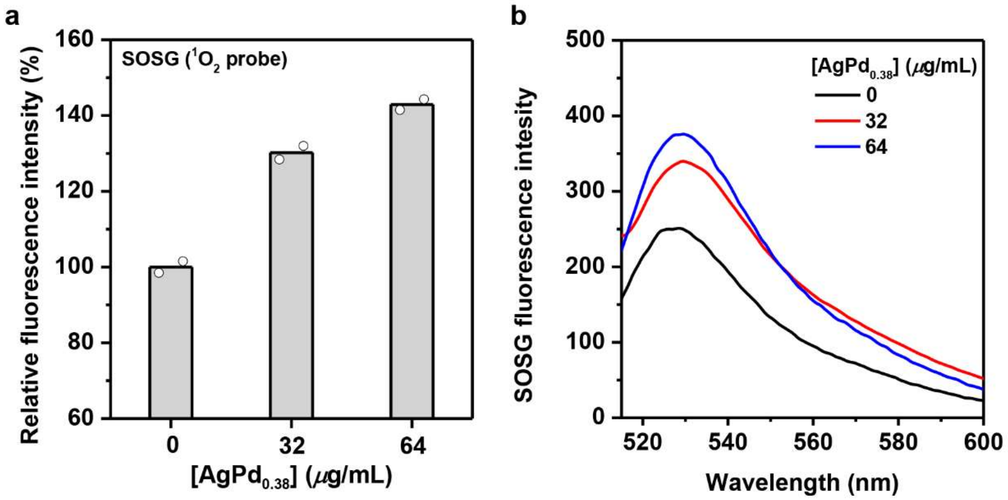

Supplementary Figure 15. Oxidation of SOSG by the ROS generated by $AgPd_{0.38}$. (a) Relative fluorescence intensity (at 525 nm) of SOSG treated with $AgPd_{0.38}$ (0, 32, or 64

μg/mL; for 3 h) to SOSG treated similarly but with PBS (*i.e.*, at 0 μg/mL $AgPd_{0.38}$). (b) Fluorescence emission spectra of SOSG after 3-h treatment with $AgPd_{0.38}$ (0, 32, or 64 μg/mL).

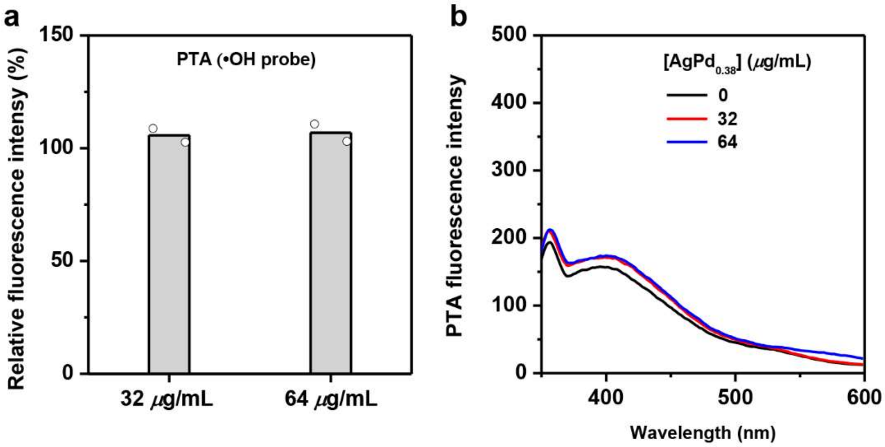

Supplementary Figure 16. The ROS generated by $AgPd_{0.38}$ are unable to oxidize PTA. (a) Relative fluorescence intensity (λ_{em} = 400 nm) of PTA treated with $AgPd_{0.38}$ (32 or 64 μg/mL, for 3 h) to PTA treated similarly but with PBS. (b) Fluorescence emission spectra (λ_{ex} =315 nm) of PTA treated with $AgPd_{0.38}$ (32 or 64 μg/mL, for 3 h), with that of PTA treated similarly but with PBS (*i.e.*, 0, μg/mL) included as a control.

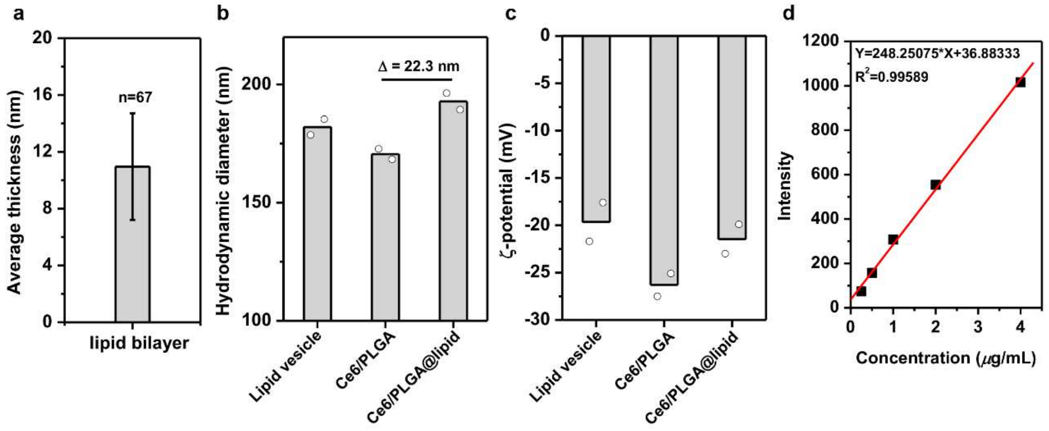

Supplementary Figure 17. Characterizations on Ce6/PLGA@lipid nanoparticles. (a) Average thickness of the lipid bilayer over Ce6/PLGA in Ce6/PLGA@lipid nanoparticles. The value of "n" denotes the number of individual nanoparticles used for extracting the average thickness of lipid bilayer. (b) Hydrodynamic diameters and (c) ζ-potentials of Ce6/PLGA before and after coating with a lipid bilayer in Millipore water, with those of the PEGylated liposome included as references. (d) The relationship of Ce6's fluorescence at 671 nm (λ_{ex} = 405 nm) versus its concention in acetone.

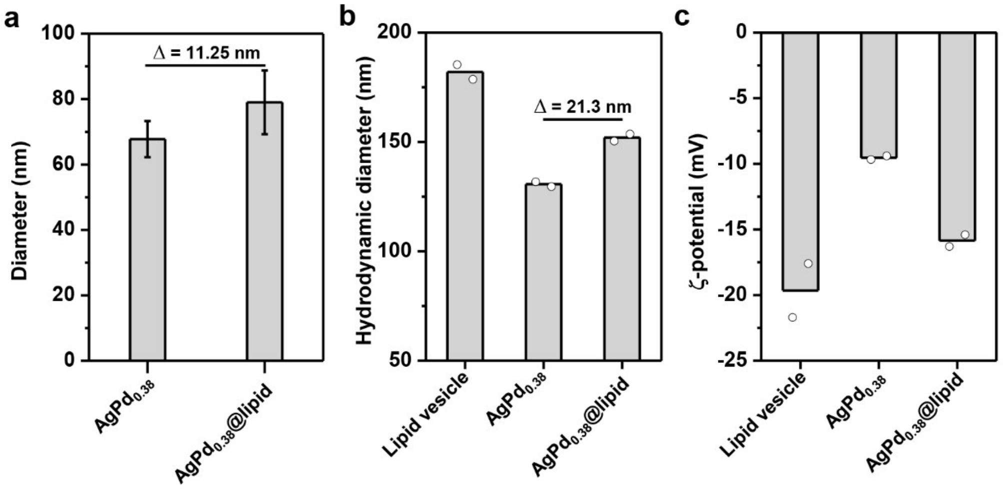

Supplementary Figure 18. Characterizations on $AgPd_{0.38}$@lipid. (a) Average size of

AgPd$_{0.38}$@lipid (over 50 individual nanoparticles) under TEM, which is 11.25 nm larger than that of AgPd$_{0.38}$ (over 50 individual nanoparticles). (b) Hydrodynamic diameters and (c) ζ-potentials of AgPd$_{0.38}$ and AgPd$_{0.38}$@lipid, with those of the precusor PEGylated liposome included for comparison.

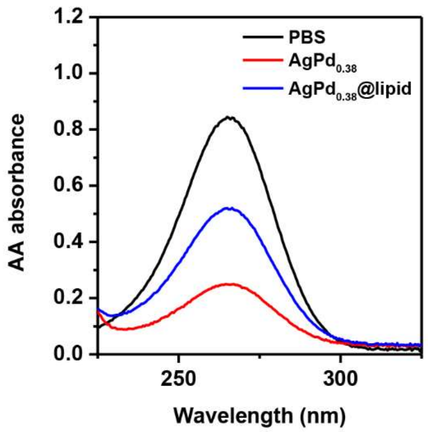

Supplementary Figure 19. Effects of lipid bilayer coating on AgPd$_{0.38}$'s ability to oxidize AA. Absorption spectrum of AA treated with AgPd$_{0.38}$@lipid (8 μg/mL in AgPd dose, for 3-h), with that of AA treated similarly but with AgPd$_{0.38}$ (8 μg/mL in AgPd dose) included for comparison. Control is AA treated similarly but with PBS.

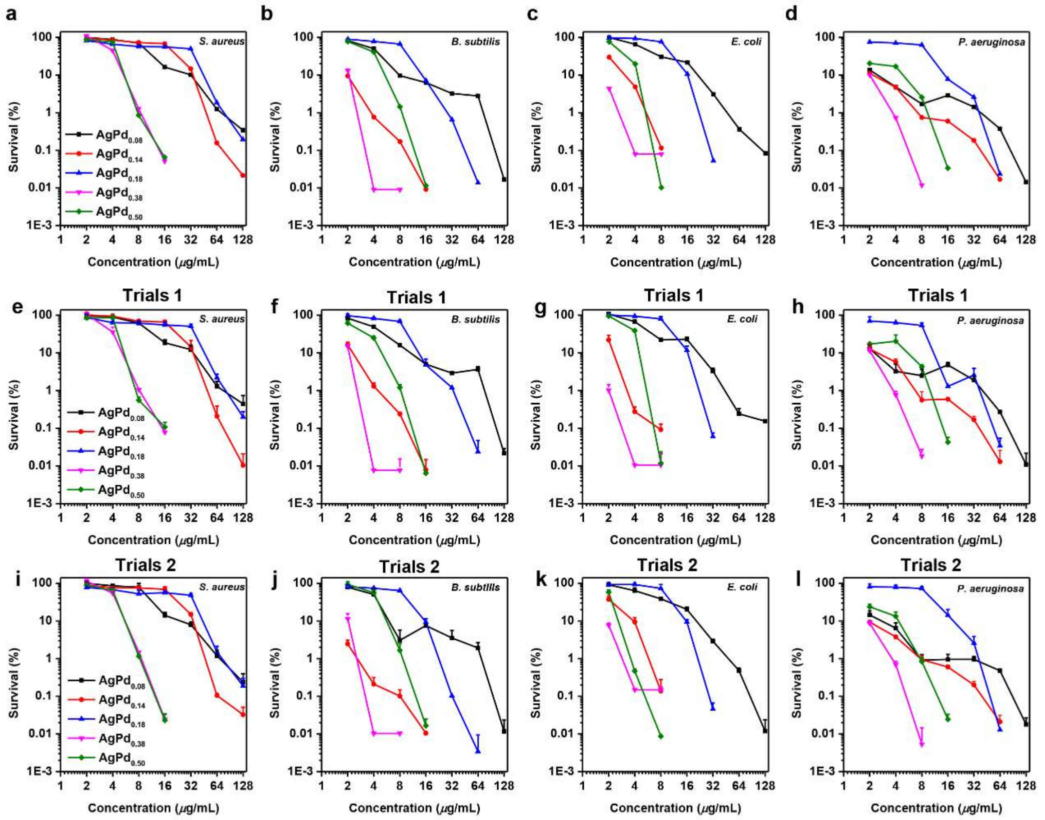

Supplementary Figure 20. *In vitro* antibacterial assays. Survival ratios of (a) *S. aureus*, (b) *B. subtilis*, (c) *E. coli*, and (d) *P. aeruginosa* after 3-h treatment with AgPd nanocages. Bactericidal potency is indicated by the extent of reduction in bacterial survival. The reported results are averages of two independent trials that are reported separately in (e-h) trials 1 and (i-l) trials 2. Data points are reported as mean ± standard deviation (n = 3).

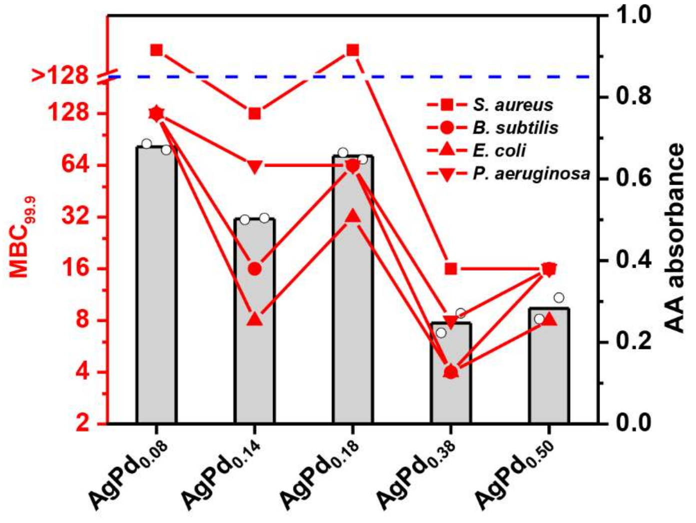

Supplementary Figure 21. (Red) The relationship between $MBC_{99.9}$ versus Pd content in AgPd nanocage, and (black) that between absorbance (λ = 266 nm) of nanocage-treated AA versus Pd content in AgPd nanocage. // indicates >128 μg/mL. The nanocage treament to AA was performed by coincubating AA with an AgPd nanocage (at 8 μg/mL) for 3 h in PBS.

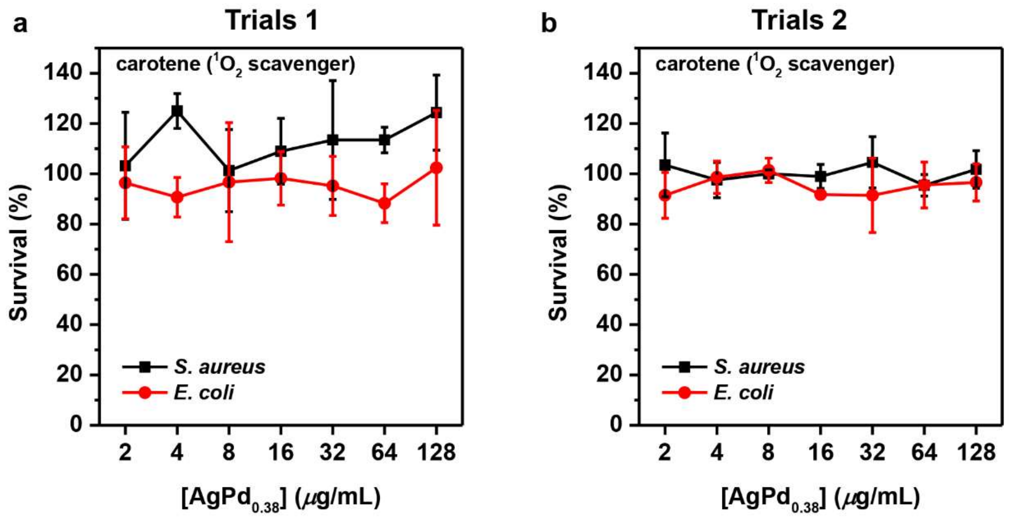

Supplementary Figure 22. Effects of carotene on $AgPd_{0.38}$'s antibacterial activity

against E. coli and S. aureus. Each plate-killing assay was carried out in triplicate and the reported results in Fig. 2b are averages of two independent trials, which are reported separately in (a) trials 1 and (b) trials 2. Data points are reported as mean ± standard deviation (n = 3).

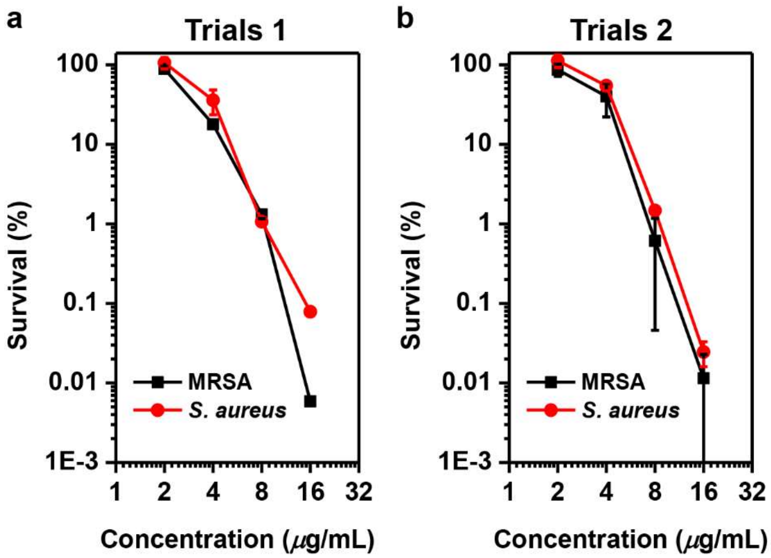

Supplementary Figure 23. Antibacterial activity of $AgPd_{0.38}$ against methicillin-resistant *Staphylococcus aureus* (MRSA) versus antibiotic-sensitive S. aureus. Each plate-killing assay was carried out in triplicate and the reported results in Fig. 2c are averages of two independent trials, which are reported separately in (a) trials 1 and (b) trials 2. Data points are reported as mean ± standard deviation (n = 3).

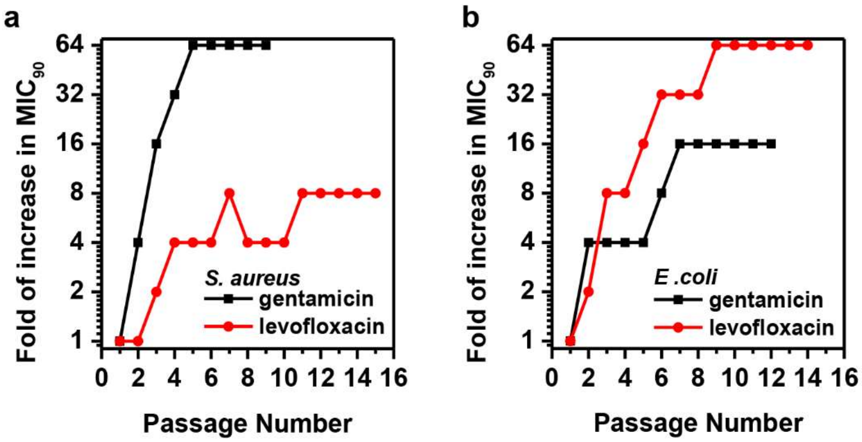

Supplementary Figure 24. Drug resistance development profiles. (a-b) Folds of increase in MIC_{90} for gentamicin and levofloxacin through serial passages of growth inhibition assays, using (a) *S. aureus* and (b) *E. coli* as the representatives for Gram-positive and –negative bacteria, respectively.

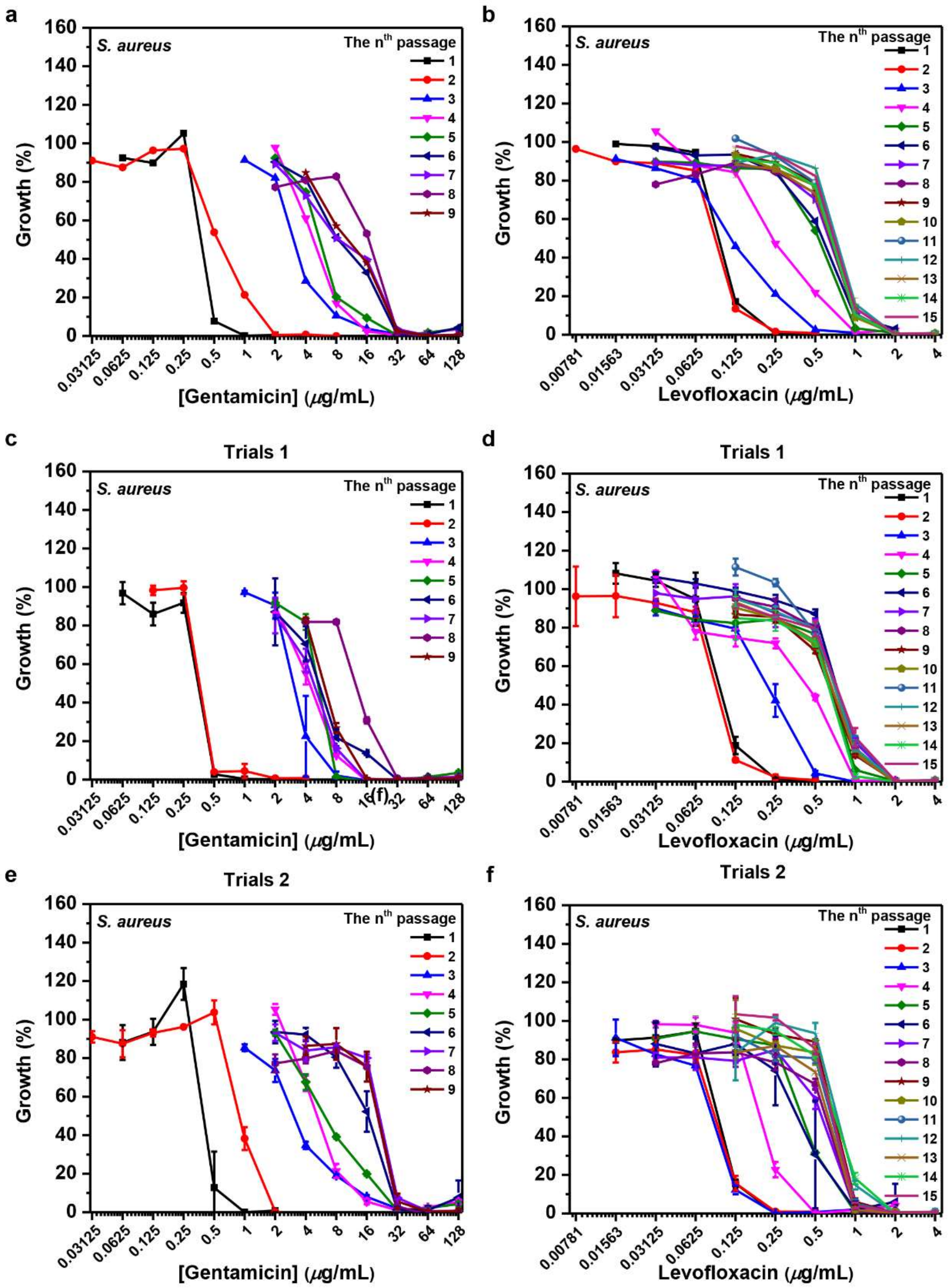

Supplementary Figure 25. Serial growth inhibition assays with antibiotics against *S. aureus*. (a-b) Growth percentages of *S. aureus* in the presence of (a) gentamicin and (b) levofloxacin in the nth passage of growth inhibition assays. Each growth inhibition assay was carried out in triplicate and (a-b) the reported results are averages of two

independent trials, which are ploted separately in (c-d) trials1 and (e-f) trials 2. Data points are reported as mean ± standard deviation (n = 3).

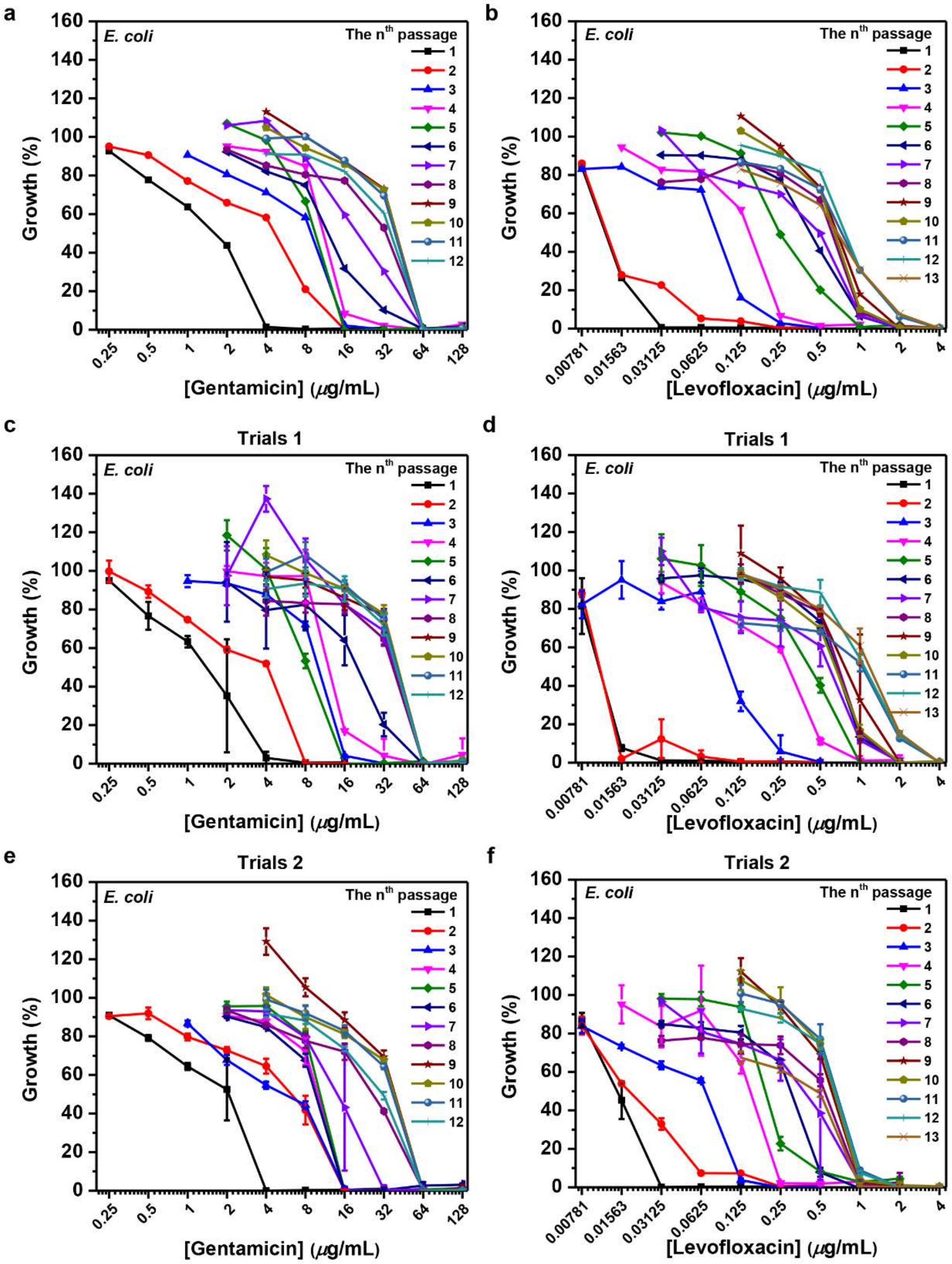

Supplementary Figure 26. Serial growth inhibition assays with antibiotics against *E.*

coli. (a-b) Growth percentages of *E. coli* in the presence of (a) gentamicin and (b) levofloxacin in the nth passage of growth inhibition assays. Each growth inhibition assay was carried out in triplicate and (a-b) the reported results are averages of two independent trials, which are ploted separately in (c-d) trials1 and (e-f) trials 2. Data points are reported as mean ± standard deviation (n = 3).

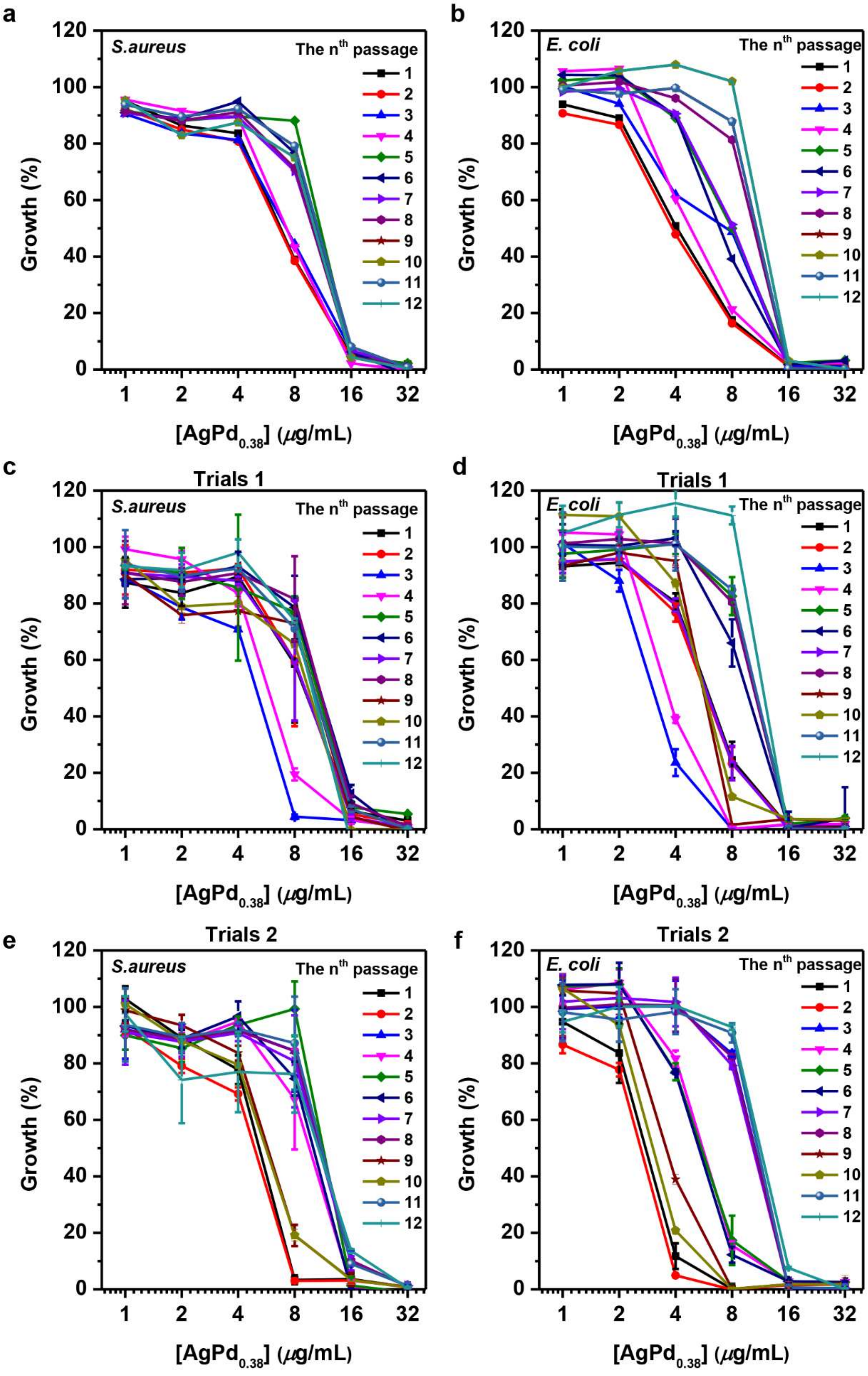

Supplementary Figure 27. Serial growth inhibition assays with $AgPd_{0.38}$. Growth

percentages of (a) *S. aureus* and (b) *E. coli* in the presence of $AgPd_{0.38}$ in the nth passage of growth inhibition assays. Each growth inhibition assay was carried out in triplicate and (a-b) the reported results are averages of two independent trials, which are ploted separately in (c-d) trials 1 and (e-f) trials 2. Data points are reported as mean ± standard deviation (n = 3).

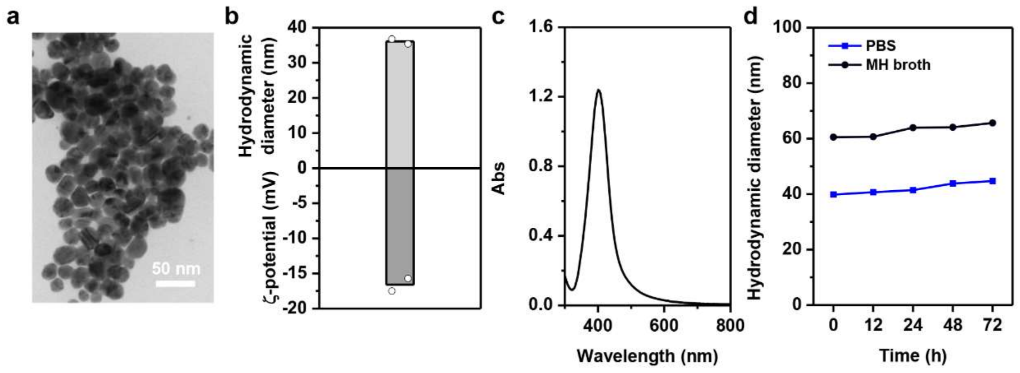

Supplementary Figure 28. Characterizations on silver nanoparticle (AgNP). (a) TEM image of AgNP. (b) Hydrodynamic diameter and ζ-potential of AgNP (10 μg/mL) in Millipore water. Data points are reported as mean ± standard deviation. (c) Absorption spectrum of AgNP dispersion (8 μg/mL) in Millipore water. (d) Hydrodynamic diameters of AgNP (10 μg/mL) in PBS and in Mueller-Hinton (MH) broth. The reported results are averages of two independent trials.

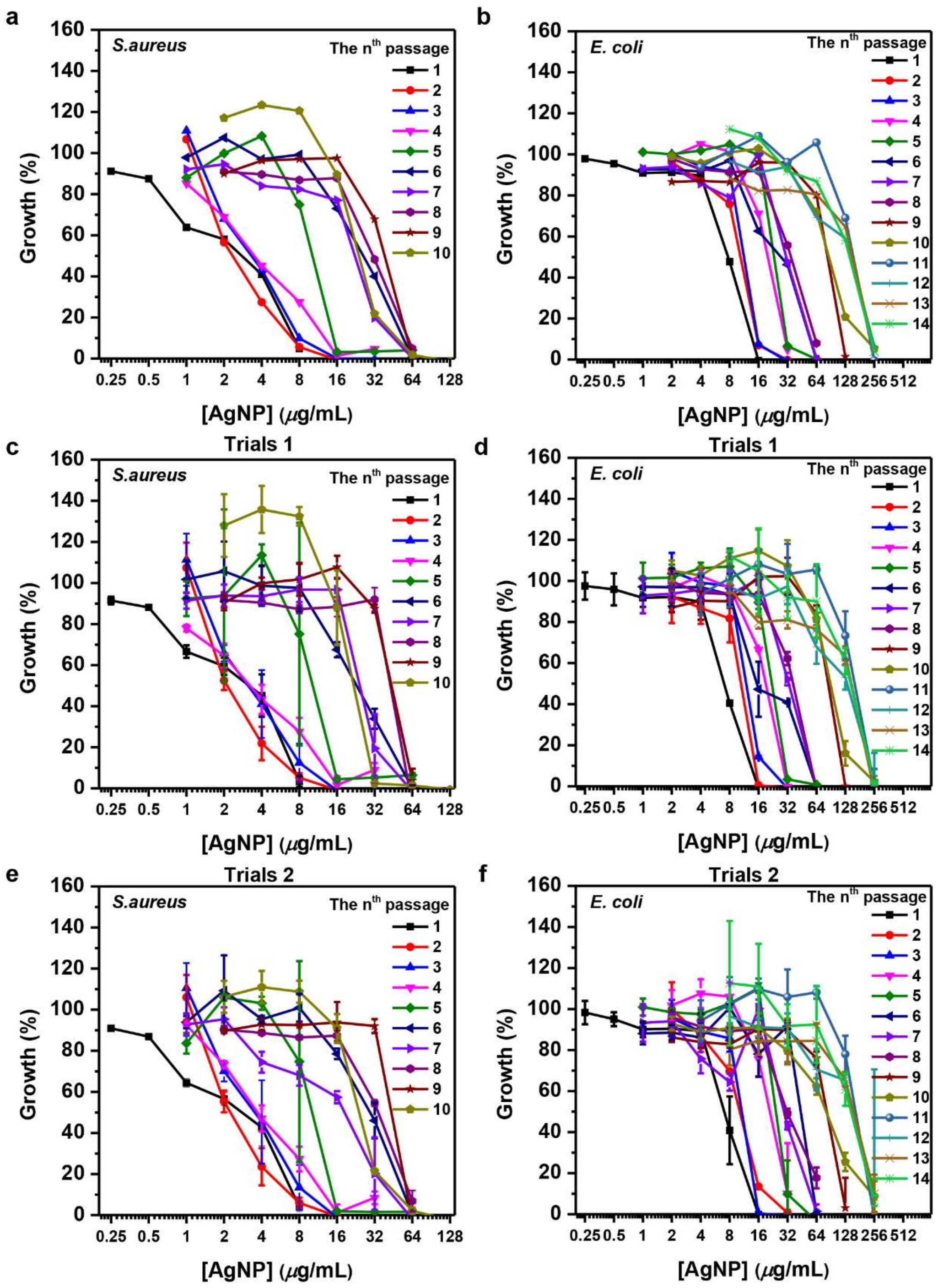

Supplementary Figure 29. Serial growth inhibition assays with AgNP. Growth percentages of (a) *S. aureus* and (b) *E. coli* in the presence of AgNP in the nth passage of growth inhibition assays. Each growth inhibition assay was carried out in triplicate and (a-b) the reported results are averages of two independent trials, which are ploted

separately in (c-d) trials 1 and (e-f) trials 2. Data points are reported as mean ± standard deviation (n = 3).

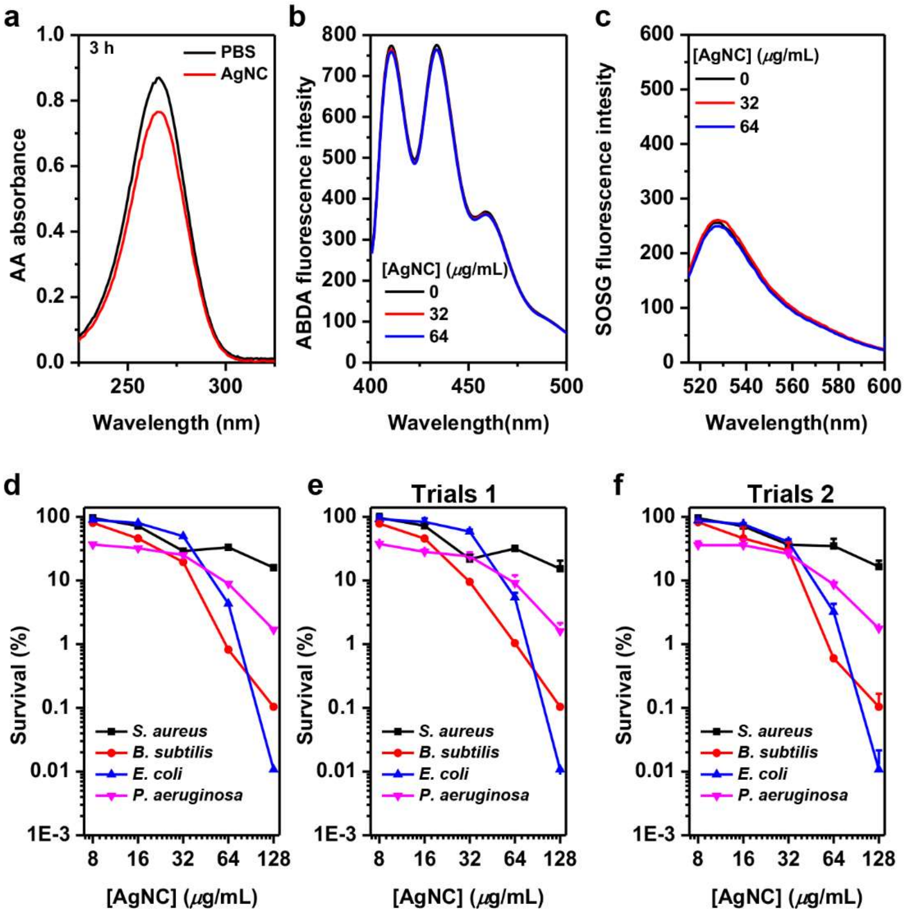

Supplementary Figure 30. Performance of AgNC. (a) Absorption spectrum of AA after 3-h treatment with AgNC (8 μg/mL) in PBS, with that of AA treated similarly but with PBS included as a control. (b) Fluorescence emission spectra of ABDA after 3-h treatment with AgNC at different concentration. (c) Fluorescence emission spectra of SOSG after 3-h treatment with AgNC at different concentration. (d) Survival ratios of bacteria (*S. aureus*, *B. subtilis*, *E. coli*, and *P. aeruginosa*) after 3-h treatment with AgNC. Each growth inhibition assay was carried out in triplicate and (d) the reported

results are averages of two independent trials, which are ploted separately in (e) trials 1 and (f) trials 2. Data points are reported as mean ± standard deviation (n = 3).

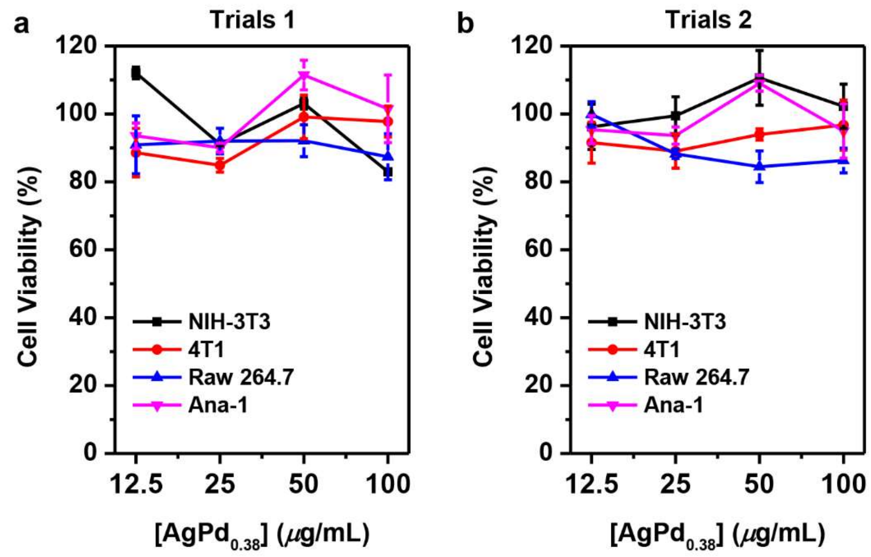

Supplementary Figure 31. Cell viability assays with $AgPd_{0.38}$. Murine macrophage Raw 264.7, macrophage Ana-1, murine embryo fibroblast NIH-3T3, and murine breast cancer 4T1 were used as representatives for mammalian cell-lines. Each cell viability assay was carried out in triplicate and the results reported in Fig. 2f are averages of two independent trials, which are ploted separately in with (a) trials 1 and (b) trials 2. Data points are reported as mean ± standard deviation (n = 3).

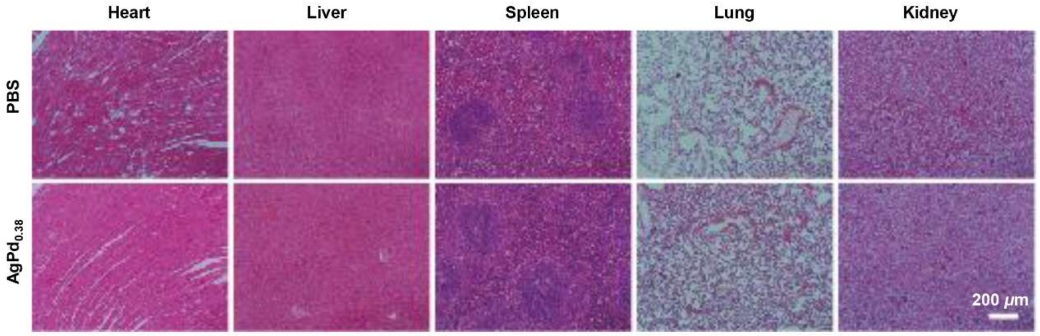

Supplementary Figure 32. H&E stained sections of major organs collected from different treatment groups on day 9.

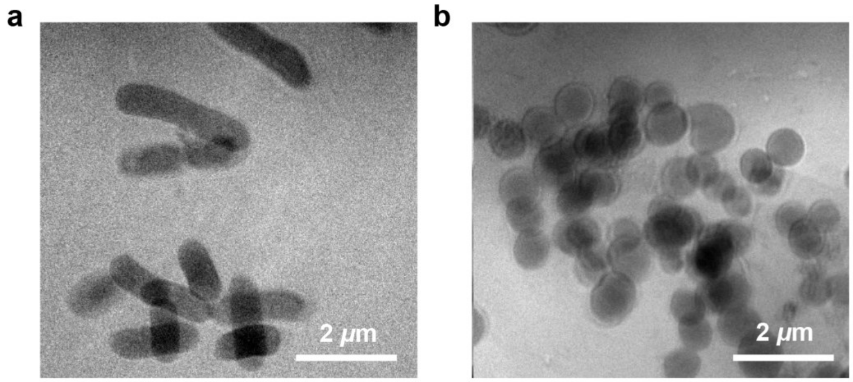

Supplementary Figure 33. Synchrotron soft X-ray microscopy images of (a) *S. aureus and* (b) *E. coli* in water.

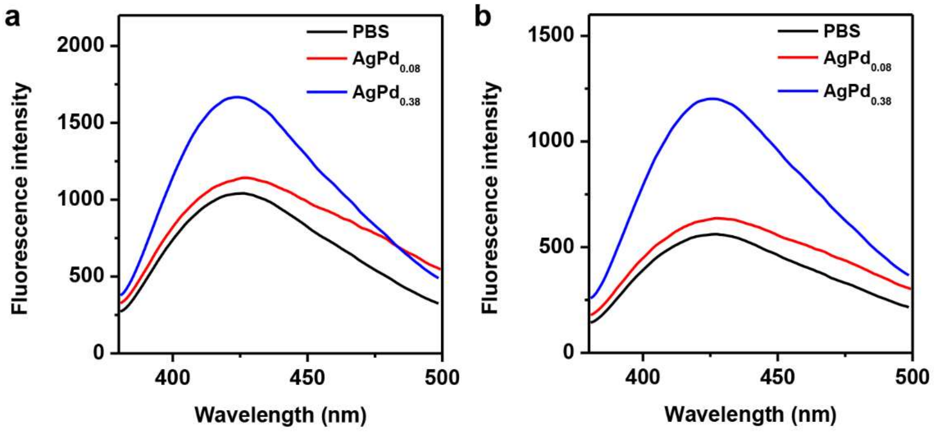

Supplementary Figure 34. (a-b) Fluorescence emission spectra of NPN (λ_{ex} = 350 nm) co-incubated (for 30 min) with nanocage-pretreated (a) *E. coli* and (b) *P. aeruginosa* cells (with 20 μg/mL nanocage, for 3 h), with those of NPN co-incubated with PBS-pretreated bacterial cells included as controls. $AgPd_{0.08}$ and $AgPd_{0.38}$ were used as the representatives for AgPd nanocages that are inactive and active against bacteria, respectively.

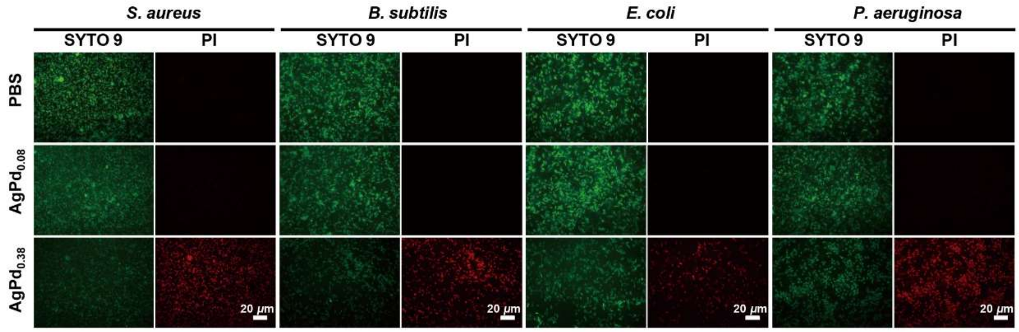

Supplementary Figure 35. Confocal fluorescence microscopy images of bacterial cells after 3-h treatment with a nanocage (20 μg/mL), with those of cells treated similarly but with PBS included as controls. SYTO 9 (green) and PI (red) were used to label all and dead bacterial cells, respectively. $AgPd_{0.08}$ and $AgPd_{0.38}$ were used as the representatives for AgPd nanocages that are inactive and potent against bacteria, respectively.

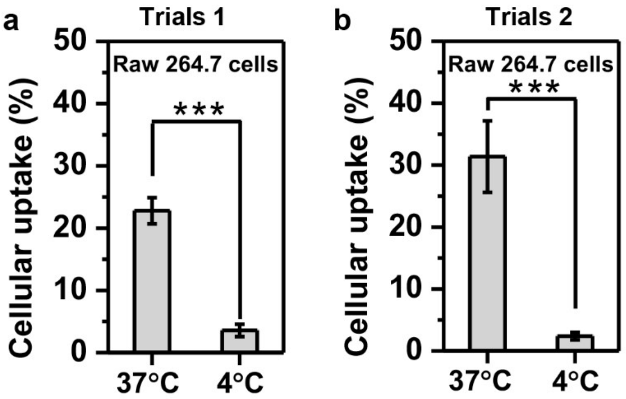

Supplementary Figure 36. Effects of temperature on the cellular uptake of $AgPd_{0.38}$. Each cellular uptake assay was carried out in triplicate and the reported results in Fig. 3c are averages of two independent trials, which are ploted separately in (a) trials 1 and (b) trials 2. Data points are reported as mean ± standard deviation (n = 3). *** indicates $p < 0.001$, analyzed by two-sided Student's t test.

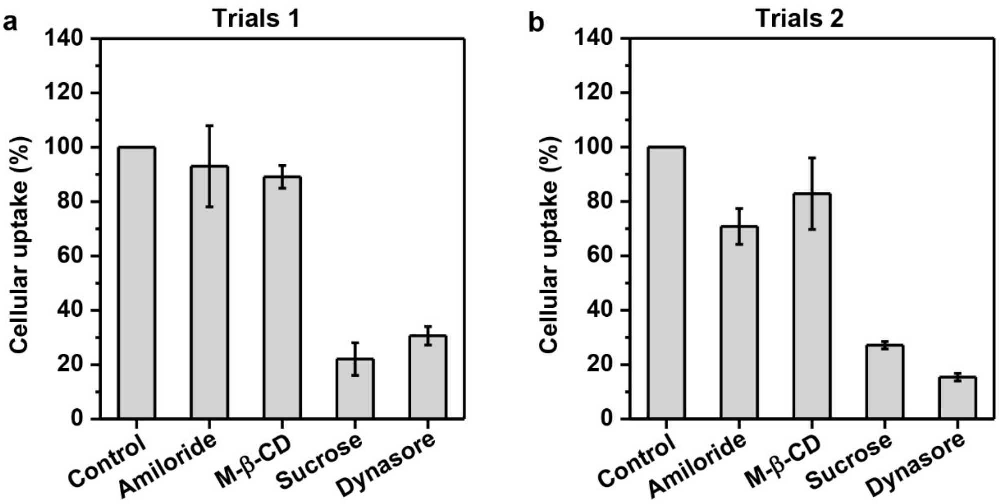

Supplementary Figure 37. Effects of endocytosis inhibitors on the cellular uptake of $AgPd_{0.38}$ at 37 ºC. Murine macrophage Raw 264.7 was used as a representative for mammalian cell lines. Each cellular uptake assay was carried out in triplicate and the reported results in Fig. 3e are averages of two independent trials, which are ploted

separately in (a) trials 1 and (b) trials 2. Data points are reported as mean ± standard deviation (n = 3).

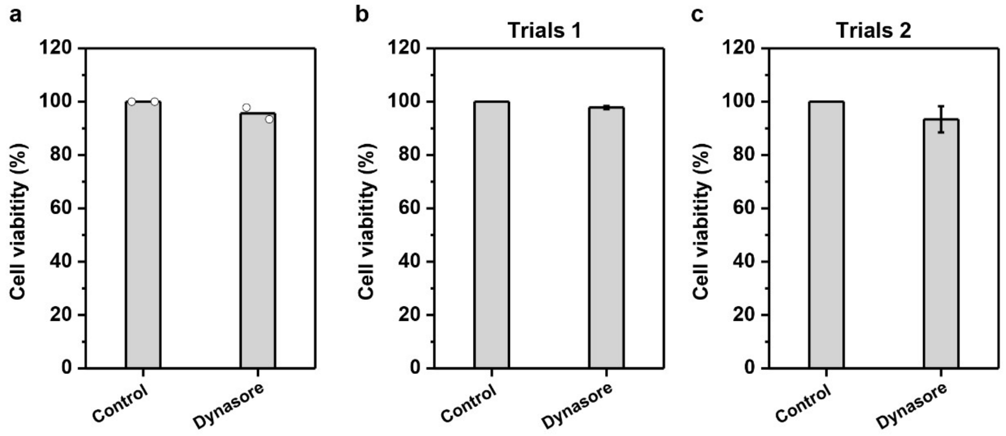

Supplementary Figure 38. Dynasore lacks intrinsic cytotoxicity. (a) Viability ratio of Raw 264.7 cells in the presence of dynasore, as shown in percentage relative to that of Raw 264.7 cells cultured similarly but in the absence of dynasore (*i.e.*, control). Each cell viability assay was carried out in triplicate and (a) the reported results are averages of two independent trials, which are ploted separately in (b) trials 1 and (c) trials 2. Data points are reported as mean ± standard deviation (n = 3).

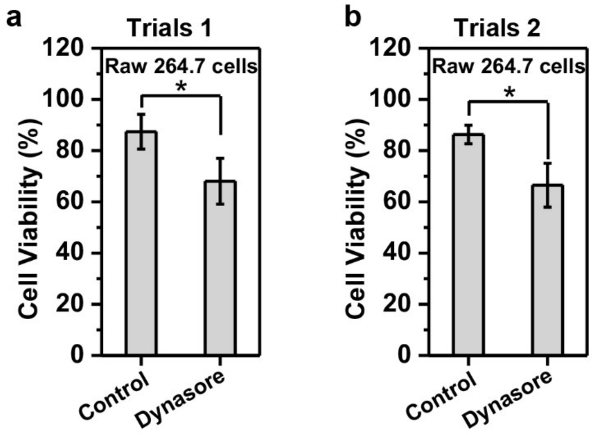

Supplementary Figure 39. Effects of dynasore on the cytotoxicity of $AgPd_{0.38}$. Viability ratios of Raw 264.7 cells treated with $AgPd_{0.38}$ (100 μg/mL) in the presence of dynasore. Controls are Raw 264.7 cells treated similarly but in the absence of dynasore. Each cell viability assay was carried out in triplicate and the reported results in Fig. 3f are averages of two independent trials, which are ploted separately in (a) trials 1 and (b) trials 2. Data points are reported as mean ± standard deviation (n = 3). * indicates $p < 0.05$, analyzed by two-sided Student's t test.

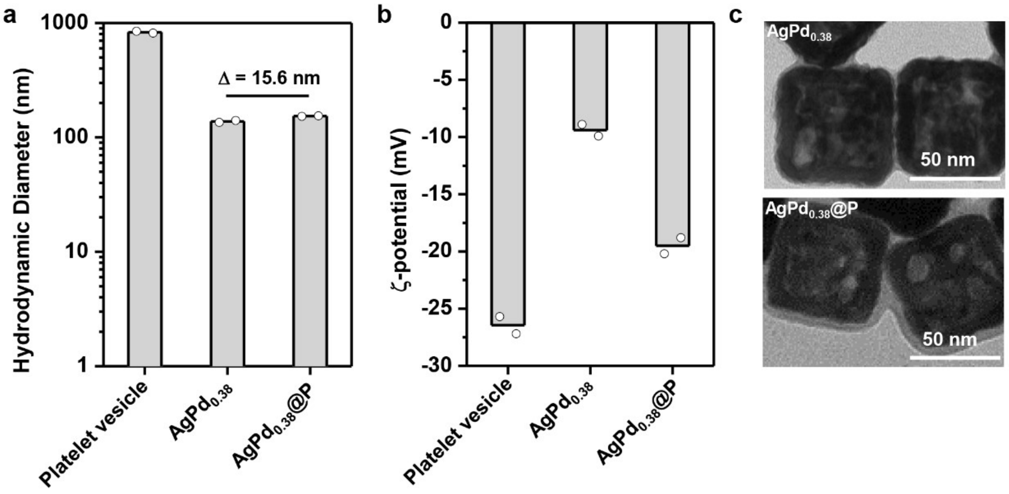

Supplementary Figure 40. Characterizations on $AgPd_{0.38}$@P. (a) Hydrodynamic diameters and (b) ζ-potentials of $AgPd_{0.38}$ before and after platelet membrane coating (*i.e.*, $AgPd_{0.38}$ versus $AgPd_{0.38}$@P), with those of the platelet membrane vesicles (*i.e.*, platelet vesicle) included for comparison. (c) TEM images of (top) $AgPd_{0.38}$ and (bottom) $AgPd_{0.38}$@P.

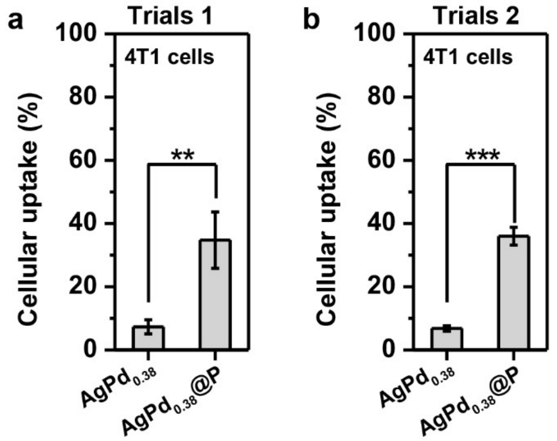

Supplementary Figure 41. Cellular uptake of $AgPd_{0.38}$@P and $AgPd_{0.38}$ by 4T1 cells. Each cellular uptake assay was carried out in triplicate and the reported results in Fig. 3h are averages of two independent trials, which are ploted separately in (a) trials 1 and (b) trials 2. Data points are reported as mean ± standard deviation (n = 3). ** indicates $p < 0.01$ and *** indicates $p < 0.001$, analyzed by two-sided Student's t test.

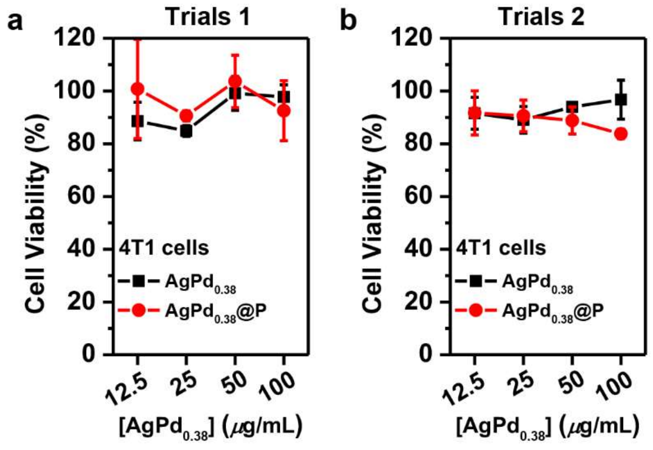

Supplementary Figure 42. Cell viability assays with $AgPd_{0.38}$@P and $AgPd_{0.38}$ to 4T1 cells. Each cell viability assay was carried out in triplicate and the reported results in Fig. 3i are averages of two independent trials, which are ploted separately in (a) trials

1 and (b) trials 2. Data points are reported as mean ± standard deviation (n = 3).

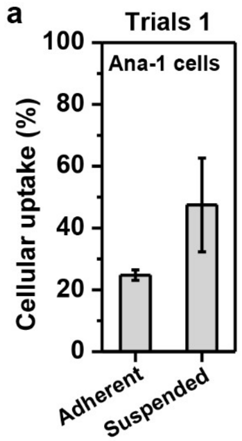

Supplementary Figure 43. Cellular uptake of $AgPd_{0.38}$ by adherent versus suspended Ana-1 cells. Each cellular uptake assay was carried out in triplicate and the reported results in Fig. 3j are averages of two independent trials, which are ploted separately in with (a) trials 1 and (b) trials 2. Data points are reported as mean ± standard deviation (n = 3). ** indicates $p < 0.01$, analyzed by two-sided Student's t test.

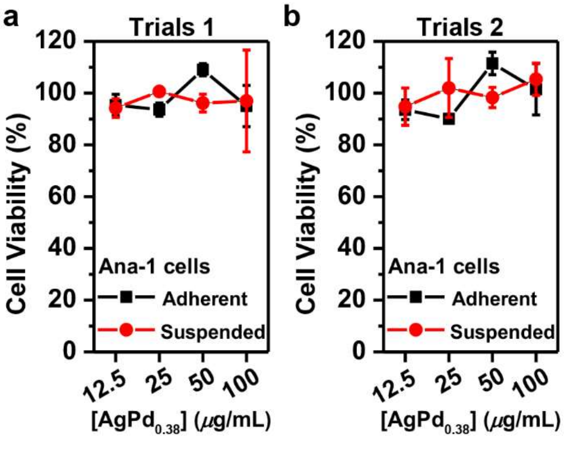

Supplementary Figure 44. Cell viability assays with $AgPd_{0.38}$ to adherent versus

suspended Ana-1 cells. Each cell viability assay was carried out in triplicate and the reported results in Fig. 3k are averages of two independent trials, which are ploted separately in with (a) trials 1 and (b) trials 2. Data points are reported as mean ± standard deviation (n = 3).

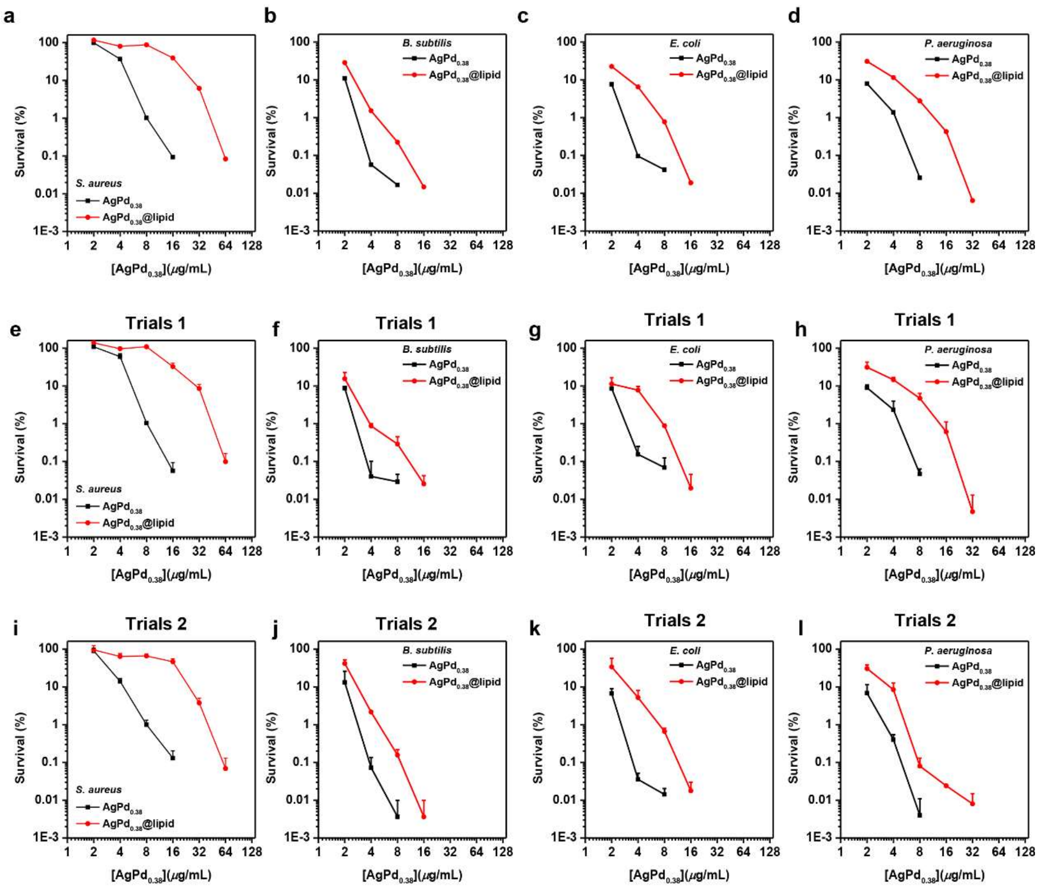

Supplementary Figure 45. Antibacterial plate-killing assays with $AgPd_{0.38}$@lipid and $AgPd_{0.38}$. Each antibacterial plate-killing assay was carried out in triplicate and (a-d) the reported results are averages of two independent trials, which are ploted separately in (e-h) trials 1 and (i-l) trials 2. Data points are reported as mean ± standard deviation (n = 3).

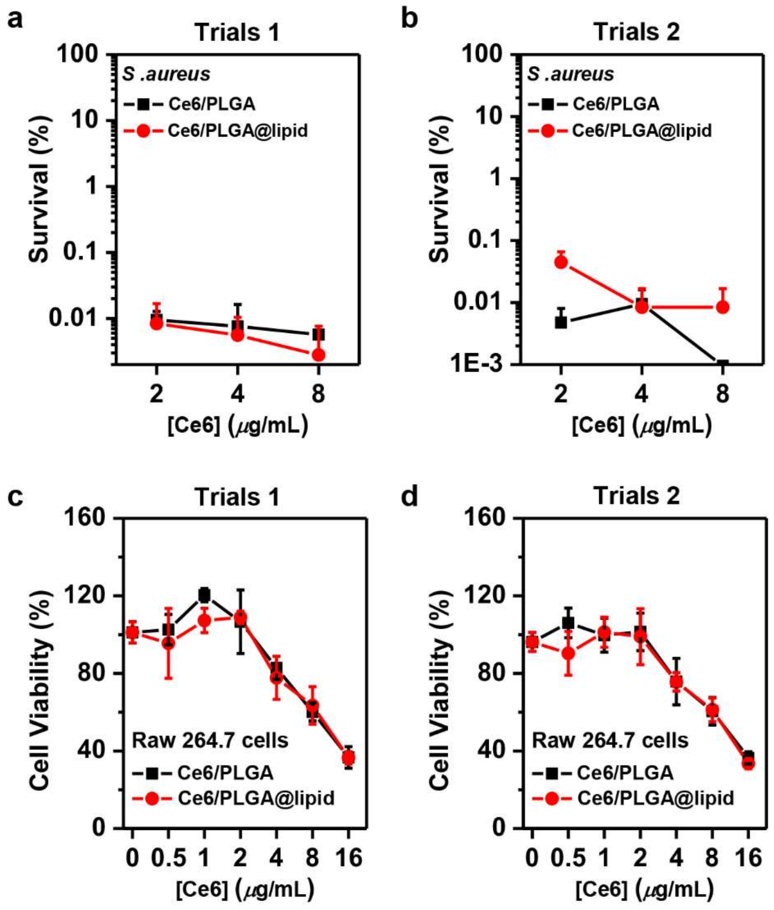

Supplementary Figure 46. Antibacterial activity and cytotoxicity of Ce6/PLGA and Ce6/PLGA@lipid. (a-b) Survival ratios of *S. aureus* after 10 min co-incubation with Ce6/PLGA or Ce6/PLGA@lipid and then 5-min irradiation (with a solar simulator at 0.1 W/m^2). Each antibacterial plate-killing assay was carried out in triplicate and the reported results in Fig. 3m are averages of two independent trials, which are ploted separately in (a) trials 1 and (b) trials 2. (c-d) Cell viability of murine macrophage Raw 264.7 cells after 4-h co-incubation with Ce6/PLGA or Ce6/PLGA@lipid at different concentrations and then 5-min irradiation (with a solar simulator at 0.1 W/m^2). Each cell viability assay was carried out in triplicate and the reported results in Fig. 3n are

averages of two independent trials, which are ploted separately in (c) trials 1 and (d) trials 2. Data points are reported as mean ± standard deviation (n = 3).

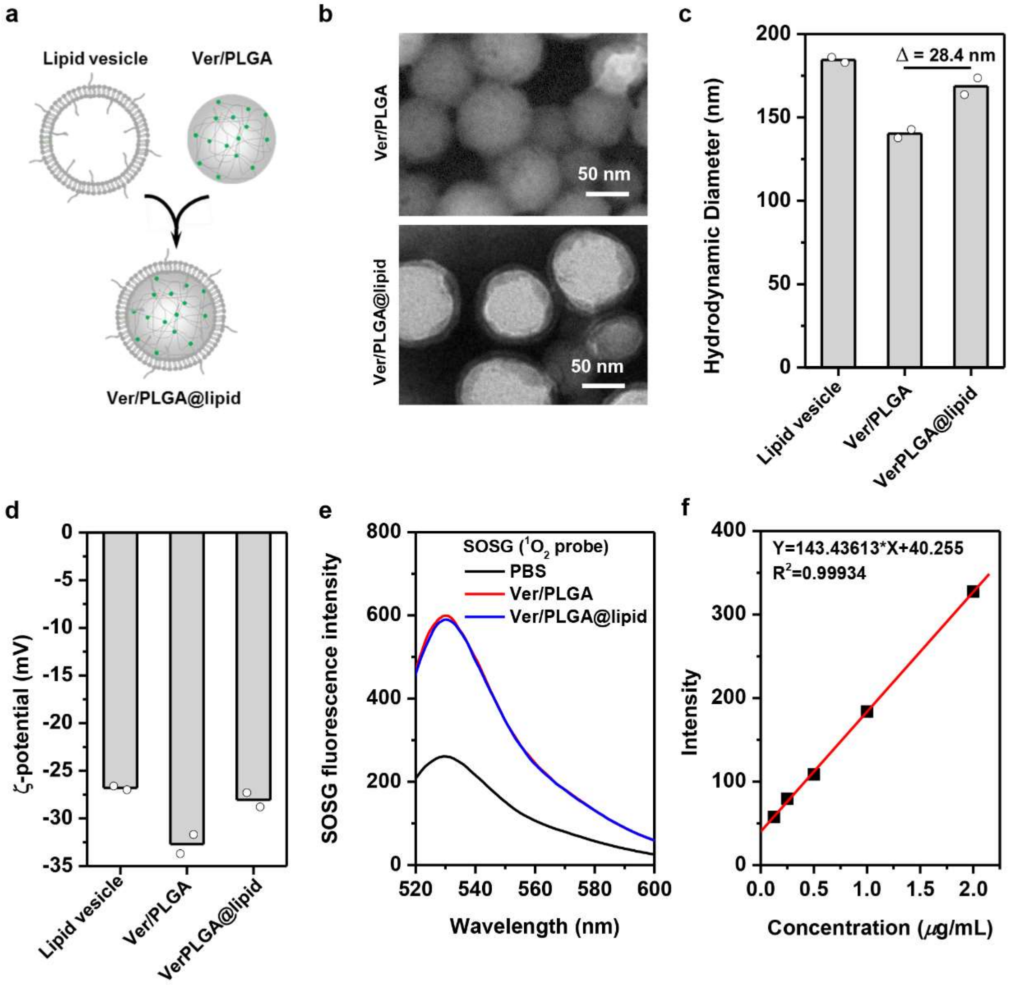

Supplementary Figure 47. Characterizations on Ver/PLGA@lipid. (a) Schematic illustration on the preparation of Ver/PLGA@lipid, by coating Ver/PLGA with a PEGylated lipid bilayer. (b) TEM images of (top) Ver/PLGA and (bottom) Ver/PLGA@lipid. (c) Hydrodynamic diameters and (d) ζ-potentials of Ver/PLGA and Ver/PLGA@lipid. (e) Fluorescence emission spectrum of SOSG treated with Ver/PLGA@lipid (at 8 μg/mL in Ver) upon irradiation with a solar simulator (at 0.1

W/m^2, 5-min), with that of SOSG treated similarly but with Ver/PLGA (at 8 μg/mL in Ver) included for comparison. Control is SOSG treated similarly but with PBS. (f) The relationship of Ver's fluorescence intensity at 690 nm (λ_{ex} = 410 nm) versus its concention in acetone.

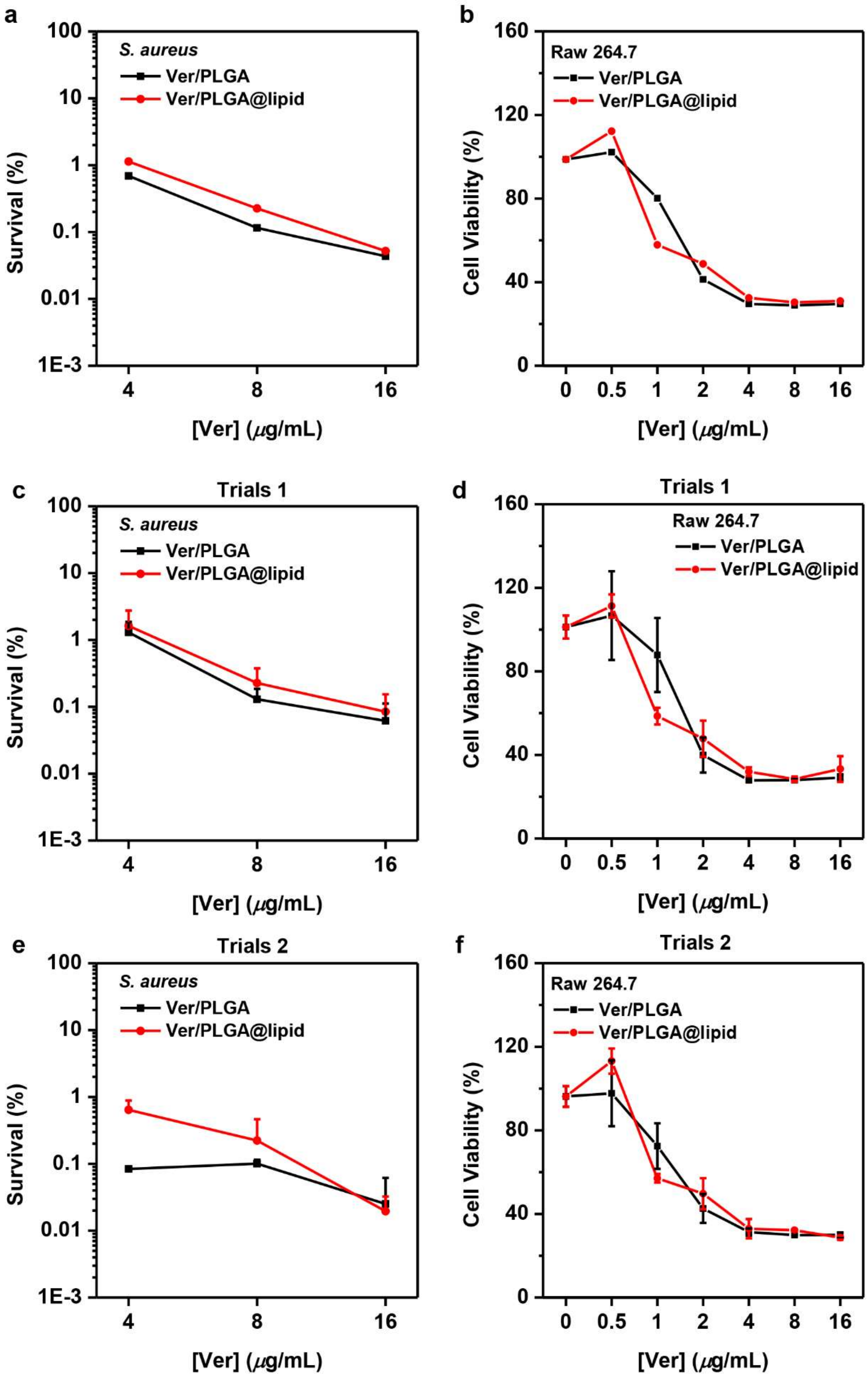

Supplementary Figure 48. Antibacterial activity and cytotoxicity of Ver/PLGA and

Ver/PLGA@lipid. (a) Survival ratios of *S. aureus* after 10 min co-incubation with Ver/PLGA or Ver/PLGA@lipid and then 5-min irradiation (with a solar simulator at 0.1 W/m^2). Each antibacterial plate-killing assay was carried out in triplicate and (a) the reported results are averages of two independent trials, which are ploted separately in (c) trials 1 and (e) trials 2. (b) Viability of murine macrophage Raw 264.7 cells after 4-h co-incubation with Ce6/PLGA or Ce6/PLGA@lipid and then 5-min irradiation (with a solar simulator at 0.1 W/m^2). Each cell viability assay was carried out in triplicate and (b) the reported results are averages of two independent trials, which are ploted separately in (d) trials 1 and (f) trials 2. Data points are reported as mean ± standard deviation (n = 3).

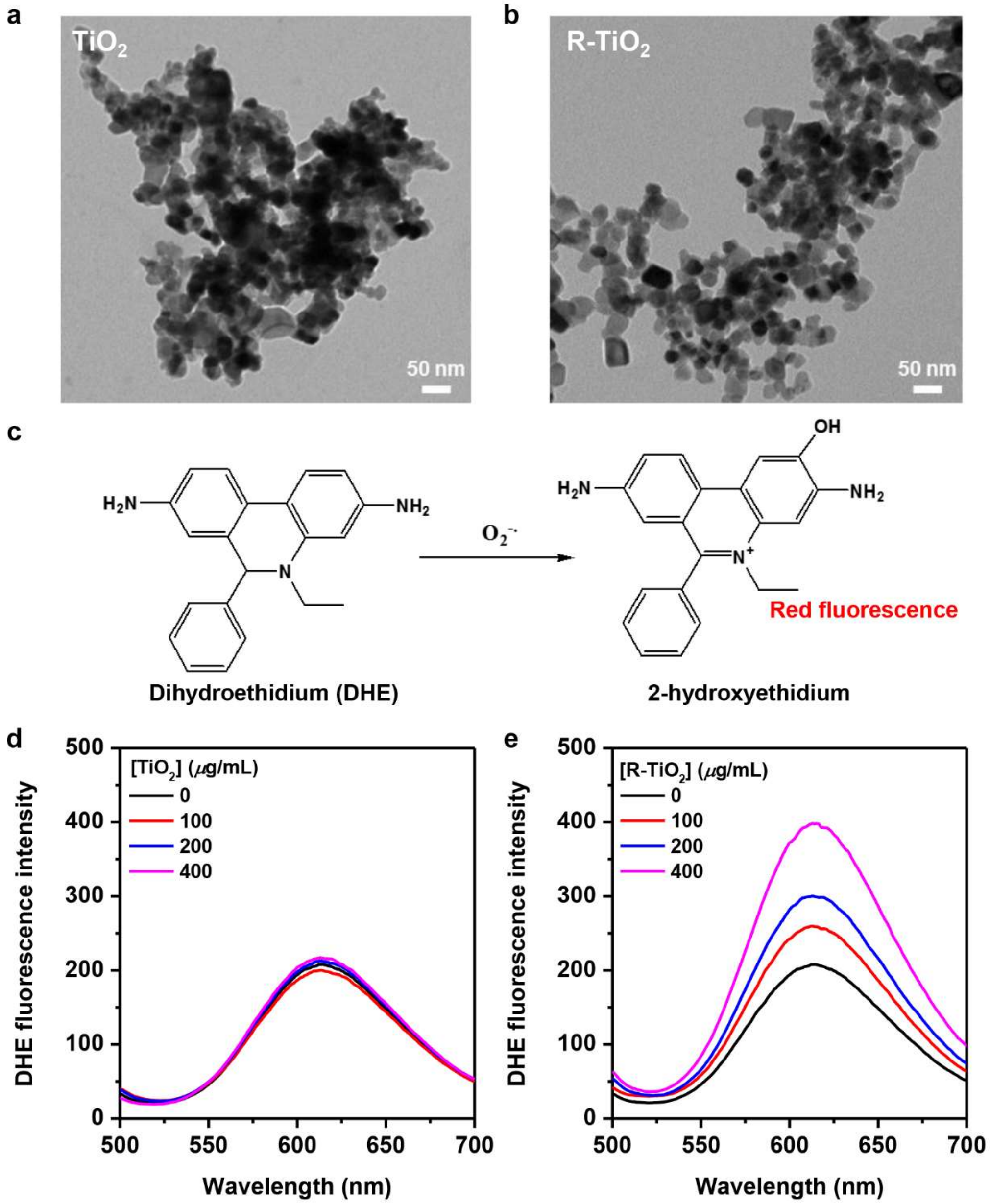

Supplementary Figure 49. Characterization on R-TiO_2. (a-b) TEM images of (a) the pristine TiO_2 nanoparticle and (b) the thermally reduced TiO_2 nanoparticle (*i.e.*, R-TiO_2). (c) Schematic illustration on the oxidation of dihydroethidium (DHE) by superoxide radical. (d-e) Fluorescence emission spectra of DHE after 3-h treatment in the dark with (d) TiO_2 and (e) R-TiO_2 at different concentration.

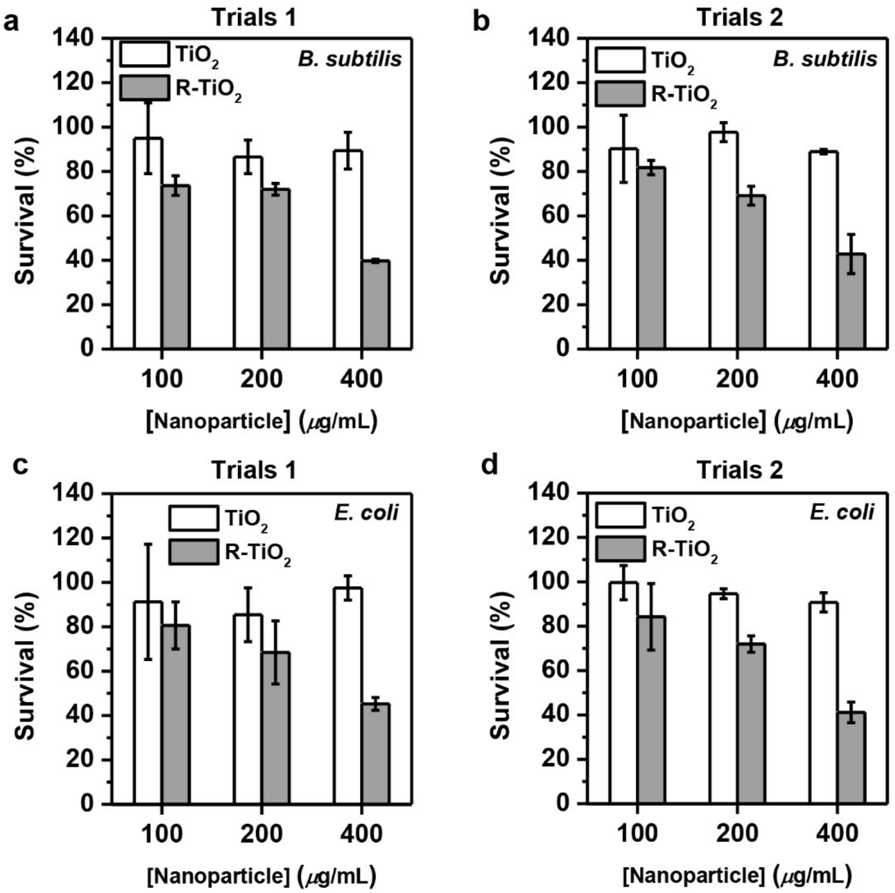

Supplementary Figure 50. Antibacterial assays with R-TiO_2 and TiO_2. (a-b) *B. subtilis* and (c-d) *E. coli* cells were used as representatives for Gram-positive and –negative bacteria, respectively. Each antibacterial assay was carried out in triplicate and the reported results in Fig. 4b-c are averages of two independent trials, which are ploted separately in (a, c) trials 1 and (b, d) trials 2. Data points are reported as mean ± standard deviation (n = 3).

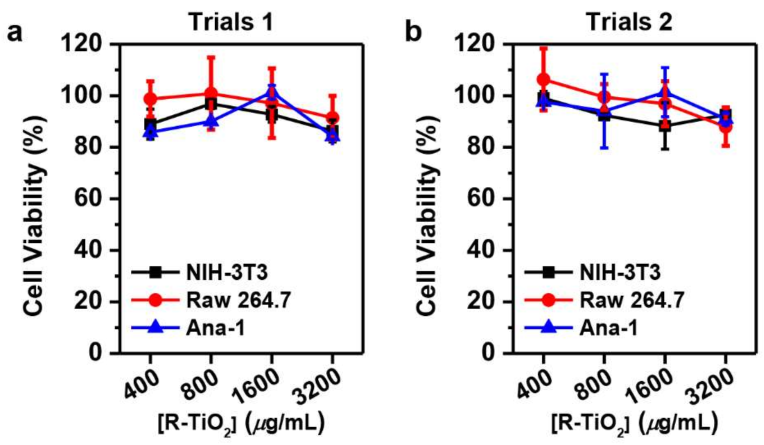

Supplementary Figure 51. Cell viability assays with R-TiO_2**.** NIH-3T3, Raw 264.7, and Ana-1 cells were used as representatives for mammalian cell lines. Each cell viability assay was carried out in triplicate and the reported results in Fig. 4d are averages of two independent trials, which are ploted separately in (a) trials 1 and (b) trials 2. Data points are reported as mean ± standard deviation (n = 3).

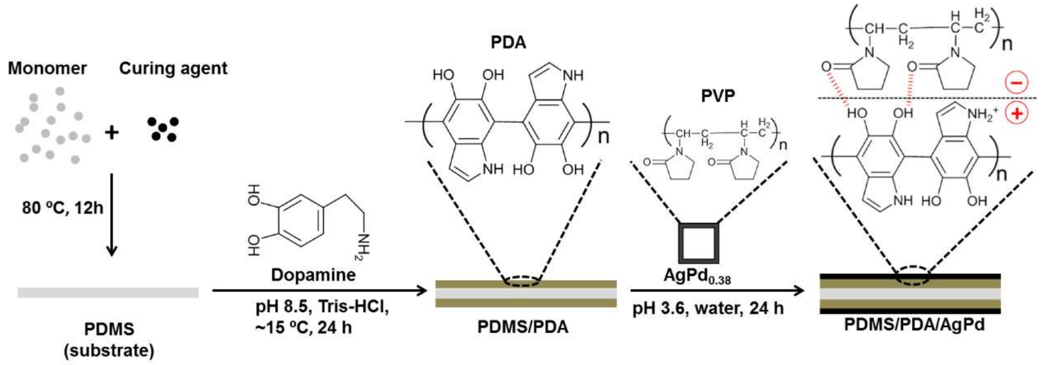

Supplementary Figure 52. Schematic illustration on the preparation of a PDMS wafer and the modifications to the resulting PDMS wafter successively with dopamine and $AgPd_{0.38}$, which yielded PDMS/PDA and PDMS/PDA/AgPd as the intermediate and final surfaces, respectively.

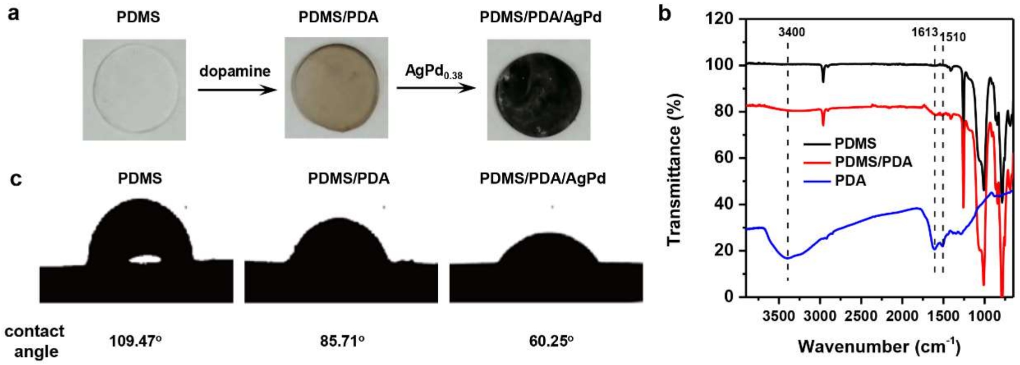

Supplementary Figure 53. Characterization of PDMS/PDA/AgPd. (a) Photographs of PDMS/PDA/AgPd, the pristine PDMS, and the intermediate PDMS/PDA. (b) Fourier Transform Infrared Spectroscopy (FT-IR) spectrum of PDMS/PDA, with those of polydopamine (PDA) and the pristine PDMS included as references. (c) Image of a drop of water on PDMS/PDA/AgPd and the coresponding contact angle. Controls are the pristine PDMS and the intermediate PDMS/PDA surfaces.

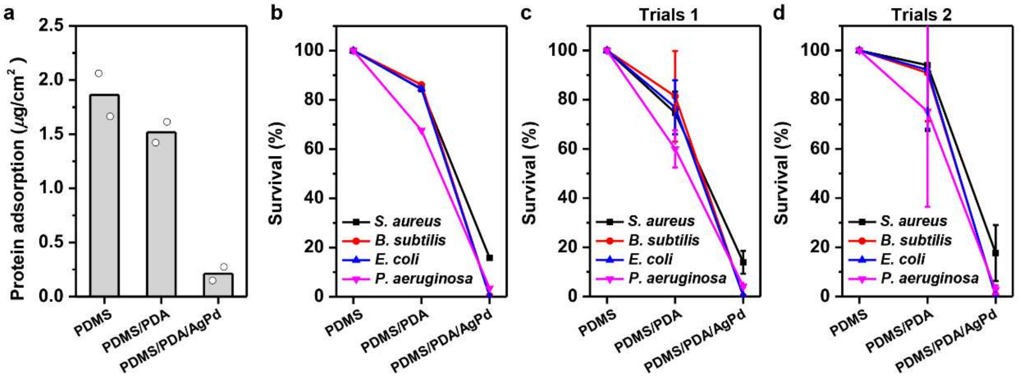

Supplementary Figure 54. Coating with $AgPd_{0.38}$ confers a PDMS substrate with antifouling and antibacterial ability. (a) Adsorption of bovin serum albumin (BSA) on PDMS/PDA/AgPd after 5-day co-incubation in water. Controls are the pristine PDMS and the intermediate PDMS/PDA. (b) Plate-killing assays of PDMS/PDA/AgPd against

planktonic bacteria. Controls are the pristine PDMS and the intermediate PDMS/PDA. Each antibacterial assay was carried out in triplicate and (b) the reported results are averages of two independent trials, which are ploted separately in (c) trials 1 and (d) trials 2. Data points are reported as mean ± standard deviation (n = 3).

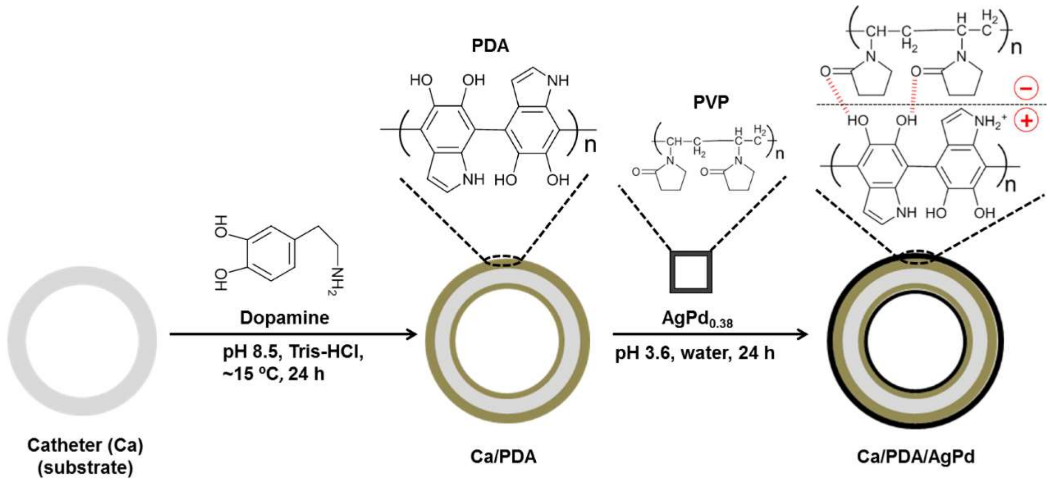

Supplementary Figure 55. Schematic illustration on the modifications to a catheter (which is readily available in clinics) successively with dopamine and $AgPd_{0.38}$, which yielded Ca/PDA and Ca/PDA/AgPd as the intermediate and final products, respectively.

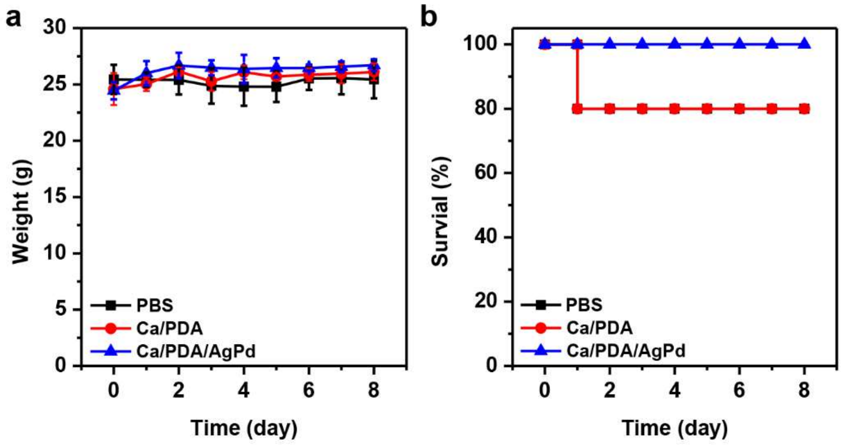

Supplementary Figure 56. (a) Average mouse weights and (b) mouse survival ratios from different treatment groups throughout the observation window. Data points are reported as mean ± standard deviation (n = 5 for all groups on day 0 and for Ca/PDA/AgPd group throughout the observation window, n = 4 for both PBS and Ca/PDA groups from day 1 through day 8).

Supplementary References

1. Winkler BS, Orselli SM, Rex TS. The redox couple between glutathione and ascorbic-acid - a chemical and physiological perspective. *Free Radical. Biol. Med.* **17**, 333-349 (1994).

2. Kim YKaM-G. Hplc-uv method for the simultaneous determinations of ascorbic acid and dehydroascorbic acid in human plasma. *Transl. Clin. Pharmacol.* **24**, 37-42 (2016).

3. Song RJ, Feng Y, Wang DH, Xu ZP, Li Z, Shao XS. Phytoalexin phenalenone derivatives inactivate mosquito larvae and root-knot nematode as type-ii photosensitizer. *Sci. Rep.* **7**, 42058 (2017).

4. Long R, Huang H, Li YP, Song L, Xiong YJ. Palladium-based nanomaterials: A platform to produce reactive oxygen species for catalyzing oxidation reactions. *Adv. Mater.* **27**, 7025-7042 (2015).

5. Wang KW*, et al.* Size-switchable nanoparticles with self-destructive and tumor penetration characteristics for site-specific phototherapy of cancer. *ACS Appl. Mater. Interfaces* **12**, 6933-6943 (2020).

6. Park H, Park W, Na K. Doxorubicin loaded singlet-oxygen producible polymeric micelle based on chlorine e6 conjugated pluronic f127 for overcoming drug resistance in cancer. *Biomaterials* **35**, 7963-7969 (2014).

7. Xue P*, et al.* Surface modification of poly(dimethylsiloxane) with polydopamine and hyaluronic acid to enhance hemocompatibility for potential applications in medical implants or devices. *ACS Appl. Mater. Interfaces* **9**, 33632-33644 (2017).

8. Jiang JH, Zhu LP, Zhu LJ, Zhang HT, Zhu BK, Xu YY. Antifouling and antimicrobial polymer membranes based on bioinspired polydopamine and strong hydrogen-bonded poly(n-vinyl pyrrolidone). *ACS Appl. Mater. Interfaces* **5**, 12895-12904 (2013).

9. Tian X*, et al.* Bactericidal effects of silver nanoparticles on lactobacilli and the underlying mechanism. *ACS Appl. Mater. Interfaces* **10**, 8443-8450 (2018).

10. Chen S, Quan Y, Yu YL, Wang JH. Graphene quantum dot/silver nanoparticle hybrids with oxidase activities for antibacterial application. *ACS Biomater. Sci. Eng.* **3**, 313-321 (2017).

11. Sun HJ, Gao N, Dong K, Ren JS, Qu XG. Graphene quantum dots-band-aids used for wound disinfection. *ACS Nano* **8**, 6202-6210 (2014).

12. Yin WY*, et al.* Functionalized nano-mos$_2$ with peroxidase catalytic and near-infrared photothermal activities for safe and synergetic wound antibacterial applications. *ACS Nano* **10**, 11000-11011 (2016).

13. Liu XP*, et al.* Two-dimensional metal-organic framework/enzyme hybrid nanocatalyst as a benign and m self-activated cascade reagent for in vivo wound healing. *ACS Nano* **13**, 5222-5230 (2019).

14. Shan JY*, et al.* Efficient bacteria killing by cu$_2$ws$_4$ nanocrystals with enzyme-like properties and bacteria-binding ability. *ACS Nano* **13**, 13797-13808 (2019).

15. Sun D*, et al.* Ultrasound-switchable nanozyme augments sonodynamic therapy against multidrug-resistant bacterial infection. *ACS Nano* **14**, 2063-2076 (2020).

16. Herget K*, et al.* Haloperoxidase mimicry by ceo$_{2-x}$ nanorods combats biofouling. *Adv. Mater.* **29**, 1603823 (2017).

17. Tao Y, Ju EG, Ren JS, Qu XG. Bifunctionalized mesoporous silica-supported gold nanoparticles: Intrinsic oxidase and peroxidase catalytic activities for antibacterial applications. *Adv. Mater.* **27**, 1097-1104 (2015).

18. Xu BL*, et al.* A single-atom nanozyme for wound disinfection applications. *Angew. Chem. Int. Edit.* **58**, 4911-4916 (2019).

19. Liu ZW, Wang FM, Ren JS, Qu XG. A series of mof/ce-based nanozymes with dual enzyme-like activity disrupting biofilms and hindering recolonization of bacteria. *Biomaterials* **208**, 21-31 (2019).

20. Wang ZZ*, et al.* Activation of biologically relevant levels of reactive oxygen species by au/g-c3n4 hybrid nanozyme for bacteria killing and wound disinfection. *Biomaterials* **113**, 145-157 (2017).

21. Xi JQ*, et al.* Copper/carbon hybrid nanozyme: Tuning catalytic activity by the copper state for antibacterial therapy. *Nano Lett.* **19**, 7645-7654 (2019).

22. Wang H*, et al.* Unraveling the enzymatic activity of oxygenated carbon nanotubes and their application in the treatment of bacterial infections. *Nano Lett.* **18**, 3344-3351 (2018).

23. Cai SF, Jia XH, Han QS, Yan XY, Yang R, Wang C. Porous pt/ag nanoparticles with excellent multifunctional enzyme mimic activities and antibacterial effects. *Nano Res.* **10**, 2056-2069 (2017).

24. Natalio F*, et al.* Vanadium pentoxide nanoparticles mimic vanadium haloperoxidases and thwart biofilm formation. *Nat. Nanotechnol.* **7**, 530-535 (2012).

25. Fang G*, et al.* Differential pd-nanocrystal facets demonstrate distinct antibacterial activity against gram-positive and gram-negative bacteria. *Nat. Commun.* **9**, 129 (2018).

26. Huo MF, Wang LY, Zhang HX, Zhang LL, Chen Y, Shi JL. Construction of single-iron-atom nanocatalysts for highly effcient catalytic antibiotics. *Small* **15**, 1901834 (2019).

27. Hu WC, Younis MR, Zhou Y, Wang C, Xia XH. In situ fabrication of ultrasmall gold nanoparticles/2d mofs hybrid as nanozyme for antibacterial therapy. *Small*

16, 2000553 (2020).

28. Zhang Y*, et al.* Nanozyme decorated metal-organic frameworks for enhanced photodynamic therapy. *ACS Nano* **12**, 651-661 (2018).

29. Zhu P, Chen Y, Shi JL. Nanoenzyme-augmented cancer sonodynamic therapy by catalytic tumor oxygenation. *ACS Nano* **12**, 3780-3795 (2018).

30. Liu F*, et al.* A tumor-microenvironment-activated nanozyme-mediated theranostic nanoreactor for imaging-guided combined tumor therapy. *Adv. Mater.* **31**, 1902885 (2019).

31. Xu BL*, et al.* Immunomodulation-enhanced nanozyme-based tumor catalytic therapy. *Adv. Mater.* **32**, 2003563 (2020).

32. Gao SS, Lin H, Zhang HX, Yao HL, Chen Y, Shi JL. Nanocatalytic tumor therapy by biomimetic dual inorganic nanozyme-catalyzed cascade reaction. *Adv. Sci.* **6**, 1801733 (2019).

33. Li SS*, et al.* A nanozyme with photo-enhanced dual enzyme-like activities for deep pancreatic cancer therapy. *Angew. Chem. Int. Edit.* **58**, 12624-12631 (2019).

34. Fu SY*, et al.* Biomimetic coo@aupt nanozyme responsive to multiple tumor microenvironmental clues for augmenting chemodynamic therapy. *Biomaterials* **257**, 120279 (2020).

35. Liu XP*, et al.* Tumor-activatable ultrasmall nanozyme generator for enhanced penetration and deep catalytic therapy. *Biomaterials* **258**, 120263 (2020).

36. Li J*, et al.* Al centre-powered graphitic nanozyme with high catalytic efficiency

for ph-independent chemodynamic therapy of cancer. *Chem. Commun.* **56**, 6285-6288 (2020).

37. Qian Liang JX, Xuejiao J. Gao, Ruofei Zhang, Yili Yang, Xingfa Gao, Xiyun Yan, Lizeng Gao, Kelong Fan A metal-free nanozyme-activated prodrug strategy for targeted tumor catalytic therapy. *Nano Today* **35**, 100935 (2020).

38. Yang Y*, et al.* Platinum-carbon-integrated nanozymes for enhanced tumor photodynamic and photothermal therapy. *Nanoscale* **12**, 13548-13557 (2020).

39. Wang DD*, et al.* Self-assembled single-atom nanozyme for enhanced photodynamic therapy treatment of tumor. *Nat. Commun.* **11**, 357 (2020).

40. Wang ZZ*, et al.* Biomimetic nanoflowers by self-assembly of nanozymes to induce intracellular oxidative damage against hypoxic tumors. *Nat. Commun.* **9**, 3334 (2018).
