# Supplementary material for: Publisher Correction: Rapid 14C excursion at 3372-3371 BCE not observed at two different locations
Source: Nat Commun. 2021 Feb 23;12:1373. doi: 10.1038/s41467-021-21647-w (PMC7902641; doi:10.1038/s41467-021-21647-w)
Supplement: Supplementary file 1 — Supplementary information [file 41467_2021_21647_MOESM1_ESM.pdf]

Supplementary Information

Supplementary Table 1. Measured values of $\Delta^{14}C$ (parts per mil) from the Debrecen (DeA) and Swiss Federal Institute of Technology (ETH) laboratories.

DeA number	Calendar age (BC)	DeA $\Delta^{14}C$	DeA error	ETH number	ETH $\Delta^{14}C$	ETH Error
	3350			ETH-91668	75.0	1.8
DeA-18968	3351	68.0	2.6	ETH-91669	71.7	2.7
DeA-20173	3352	76.4	2.8	ETH-91670	77.3	2.2
DeA-20174	3353	70.3	2.8	ETH-91671	72.3	2.6
DeA-20175	3354	74.4	2.8	ETH-91672	71.6	2.6
DeA-20176	3355	71.3	2.8	ETH-91673	73.4	2.2
DeA-20177	3356	73.3	2.8	ETH-91674	68.9	2.5
DeA-20178	3357	70.5	2.7	ETH-91675	69.0	2.6
DeA-20179	3358	72.8	2.7	ETH-91676	71.3	2.2
DeA-20180	3359	71.0	2.9	ETH-91677	71.6	2.5
DeA-20181	3360	70.5	2.8	ETH-91678	67.8	2.6
DeA-20182	3361	67.5	2.7	ETH-91679	71.1	2.2
DeA-20183	3362	69.2	2.7	ETH-91680	68.7	2.5
DeA-20184	3363	69.2	3.6	ETH-91681	68.9	2.5
DeA-20185	3364	70.8	3.8	ETH-91682	69.8	2.2
DeA-20186	3365	69.9	3.6	ETH-91683	68.6	2.5
DeA-20187	3366	69.9	2.3	ETH-91684	72.2	2.5
DeA-20262	3367	66.7	2.4	ETH-91685	69.8	2.2
DeA-20263	3368	69.1	2.5	ETH-91686	65.8	2.6
DeA-20264	3369	69.3	2.5	ETH-91687	66.9	2.5
DeA-20265	3370	65.4	2.5	ETH-91688	67.0	2.2
DeA-20266	3371	62.8	2.8	ETH-91689	61.8	2.6
DeA-20267	3372	63.2	2.6	ETH-91690	62.9	2.6
DeA-20268	3373	63.0	2.5	ETH-91691	63.1	2.2
DeA-20269	3374	62.3	2.5	ETH-91692	63.3	2.6
DeA-20270	3375	58.1	3.3	ETH-91693	64.5	2.5
DeA-20271	3376	59.3	2.8	ETH-91694	60.3	2.2
DeA-20272	3377	61.8	3.1	ETH-91695	62.9	2.4
DeA-20273	3378	60.8	2.8	ETH-91696	60.7	2.4
DeA-20274	3379	61.9	3.0	ETH-91697	62.4	2.2
DeA-20275	3380	59.5	2.8	ETH-91698	64.7	2.4
DeA-20276	3381	61.0	2.8	ETH-91699	66.6	2.4
DeA-20910	3382	62.5	2.4	ETH-91700	62.5	2.1
DeA-20911	3383	61.4	2.4	ETH-91701	62.3	2.4

DeA-20912	3384	61.6	2.4	ETH-91702	61.1	2.4
DeA-20913	3385	59.7	3.5	ETH-91703	61.5	2.2
DeA-20914	3386	57.3	2.4	ETH-91704	60.7	2.4
DeA-20915	3387	56.5	3.7	ETH-91705	59.5	2.4
DeA-20916	3388	53.8	2.5	ETH-91706	58.6	2.2
DeA-20917	3389	54.0	2.4	ETH-91707	59.1	2.4
DeA-20918	3390	59.4	2.1	ETH-91708	56.6	2.4
DeA-20919	3391	55.6	4.8			
DeA-20920	3392	56.7	2.1			

Supplementary Figure 1. Wavelet analysis of the European Oak ^{14}C series. Shades denote the dimensionless continuous wavelet transform power-spectrum density. The color bar placed on the right represents the squared power of the wavelet transform and the numbers indicate the spectrum power level (dimensionless). High values in a few time intervals (x-axis) with some periodicity (y-axis) show that the amplitude of a signal with that periodicity emerges from noise in those years. The black contour denotes the 5% significance level against red noise. The area where edge effect influence distorts the picture is shown in lighter shades. The signal attributable to the solar cycle is marked with yellow fields.

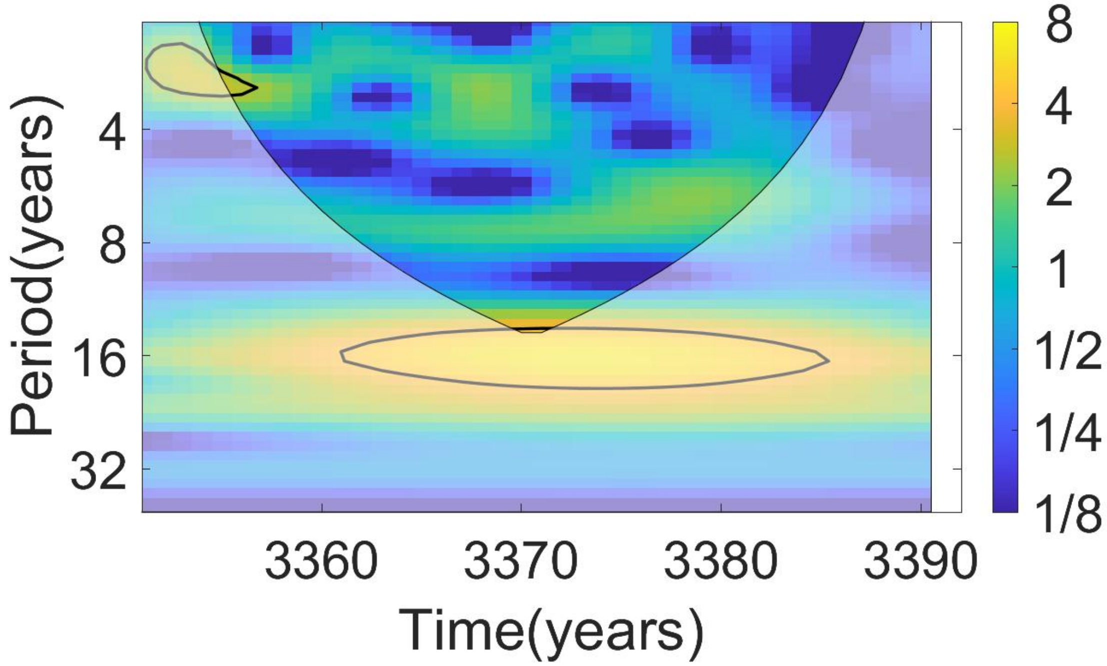

Supplementary Figure 2. Comparison of bristlecone pine sample with Chinese wingnut. a. Bristlecone pine (*Pinus longaeva*) specimen SH146-2003 used in this study for cross-dating and sampling the tree rings and b. Chinese wingnut (*Pterocarya stenoptera*) used by Wang et al. [1].

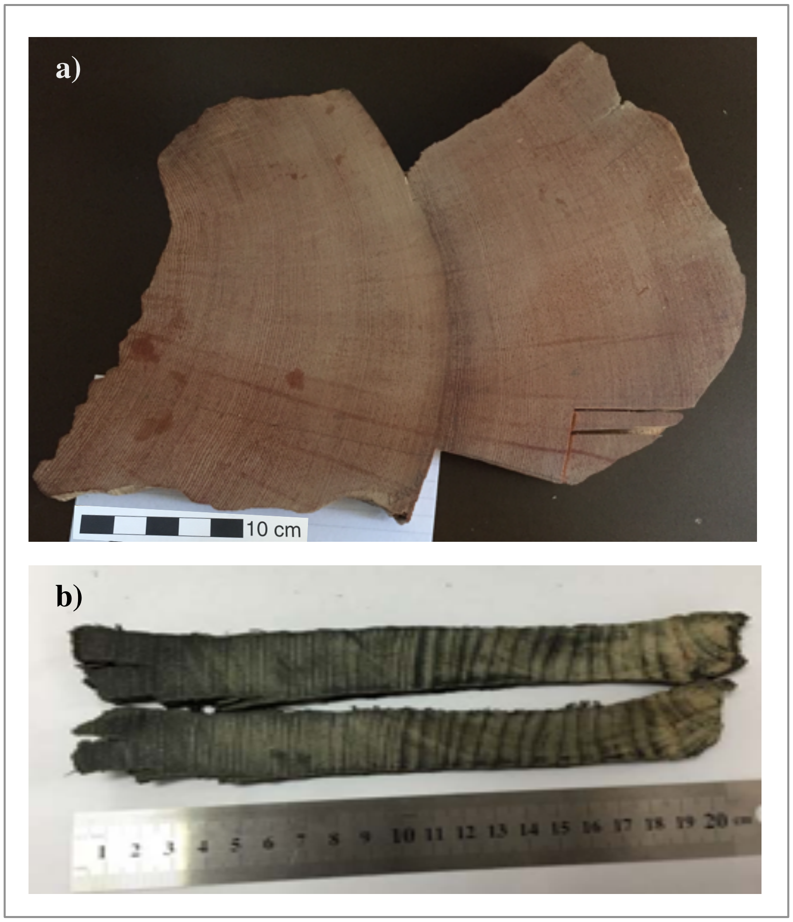

Supplementary Figure 3. Subfossil oak log excavated in 2001 from a gravel pit near in the MoselleRiver Valley in eastern France (photo W. Tegel).

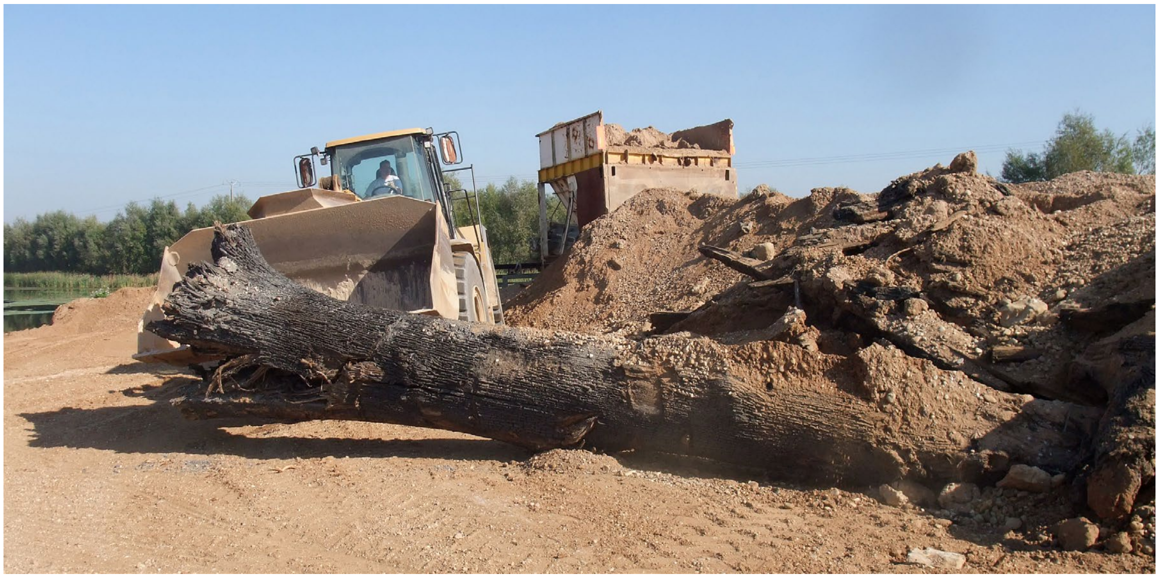

Supplementary Reference:

[1] Wang, F. Y., H. Yu, Y.C. Zou, Z.G. Dai, and K.S. Cheng A rapid cosmic-ray increase in BC 3372–3371 from ancient buried tree rings in China, Nat. Commun 8:1487, (2017) DOI: 10.1038/s41467-017-01698-8.
